# Alternative Splicing of Putative Stroke/Vascular Risk Factor Genes Expressed in Blood Following Ischemic Stroke Is Sexually Dimorphic and Cause-Specific

**DOI:** 10.3389/fneur.2020.584695

**Published:** 2020-10-22

**Authors:** Cheryl Dykstra-Aiello, Frank R. Sharp, Glen C. Jickling, Heather Hull, Farah Hamade, Natasha Shroff, Marc Durocher, Xiyuan Cheng, Xinhua Zhan, DaZhi Liu, Bradley P. Ander, Boryana S. Stamova

**Affiliations:** Department of Neurology, Medical Investigation of Neurodevelopmental Disorders (MIND) Institute Biosciences Building, University of California, Davis, Sacramento, CA, United States

**Keywords:** gene, exon, stroke, sex, etiology, array

## Abstract

Genome-wide association studies have identified putative ischemic stroke risk genes, yet, their expression after stroke is unexplored in spite of growing interest in elucidating their specific role and identifying candidate genes for stroke treatment. Thus, we took an exploratory approach to investigate sexual dimorphism, alternative splicing, and etiology in putative risk gene expression in blood following cardioembolic, atherosclerotic large vessel disease and small vessel disease/lacunar causes of ischemic stroke in each sex compared to controls. Whole transcriptome arrays assessed 71 putative stroke/vascular risk factor genes for blood RNA expression at gene-, exon-, and alternative splicing-levels. Male (*n* = 122) and female (*n* = 123) stroke and control volunteers from three university medical centers were matched for race, age, vascular risk factors, and blood draw time since stroke onset. Exclusion criteria included: previous stroke, drug abuse, subarachnoid or intracerebral hemorrhage, hemorrhagic transformation, infection, dialysis, cancer, hematological abnormalities, thrombolytics, anticoagulants or immunosuppressants. Significant differential gene expression (fold change > |1.2|, *p* < 0.05, partial correlation > |0.4|) and alternative splicing (false discovery rate *p* < 0.3) were assessed. At gene level, few were differentially expressed: ALDH2, ALOX5AP, F13A1, and IMPA2 (males, all stroke); ITGB3 (females, cardioembolic); ADD1 (males, atherosclerotic); F13A1, IMPA2 (males, lacunar); and WNK1 (females, lacunar). GP1BA and ITGA2B were alternatively spliced in both sexes (all patients vs. controls). Six genes in males, five in females, were alternatively spliced in all stroke compared to controls. Alternative splicing and exon-level analyses associated many genes with specific etiology in either sex. Of 71 genes, 70 had differential exon-level expression in stroke patients compared to control subjects. Among stroke patients, 24 genes represented by differentially expressed exons were male-specific, six were common between sexes, and two were female-specific. In lacunar stroke, expression of 19 differentially expressed exons representing six genes (ADD1, NINJ2, PCSK9, PEMT, SMARCA4, WNK1) decreased in males and increased in females. Results demonstrate alternative splicing and sexually dimorphic expression of most putative risk genes in stroke patients' blood. Since expression was also often cause-specific, sex, and etiology are factors to consider in stroke treatment trials and genetic association studies as society trends toward more personalized medicine.

## Introduction

Genome-wide association studies (GWAS) have identified many loci associated with vascular risk factors (VRFs) and ischemic stroke (IS) risk, including some stroke-cause specific genes such as alpha 1-3-glactosyl-transferase (ABO), which is suggested to be a risk gene for large vessel disease (LVD) and cardioembolism (CE) causes of IS (1), yet whether these genes also play a direct role in post-stroke pathology which might identify them as potential targets for stroke treatment, is not well-understood. Sexual dimorphism exists for etiology, pathology, outcome, and IS risk factors (2–6). This has led to the development of male- and female-specific clinical guidelines (7) and prediction models (8). However, GWAS-identified risk loci have only recently been examined for sex differences and higher heritability has been reported in women (9, 10). These studies also found differing heritability between stroke causes and a recent study reported overlap in risk loci between ischemic subtypes and hemorrhagic strokes (11). However, there are relatively few loci that are consistently associated with stroke. Additionally, we have reported differential alternative gene splicing in stroke patients compared to control subjects (12, 13), thus raising questions about whether risk loci may also be alternatively spliced and/or specific for sex or cause of stroke. To partially address these issues, we examined peripheral blood of male and female patients with specific causes of ischemic stroke at gene- and transcript- levels to determine sexual dimorphic expression, stroke cause specific expression, and alternative splicing of stroke/vascular risk factor genes compared to vascular risk factor matched control subjects.

Gene expression measurement has been revolutionized by technologies that allow assessment at whole transcript (gene), exon, and alternatively spliced transcript levels. Alternative splicing (AS) is the process of exon inclusion or exclusion from final mRNA transcripts that allows a gene to produce several isoforms with cell- and tissue-specific functions (14–20). AS and its role in transcript diversity is critical for disease susceptibility and severity and is a potential target for treatment (21–23). Indeed, differential AS has recently been reported following stroke (13), yet it has not been examined with respect to GWAS-implicated stroke or VRF risk genes and/or sex-associated differences in stroke. Therefore, we hypothesized that differential expression and AS of GWAS-identified IS and VRF genes in patients' blood following ischemic stroke is sexually dimorphic and stroke cause-specific when compared to control subjects.

## Methods

### Derivation of Risk Genes List

A PubMed search was conducted in December 2015 and May 2016 using terms “GWAS” “stroke” “risk” and “genes” to determine those genes that had been identified by genome wide association studies as having an associated stroke risk. Additionally, the Online Mendelian Inheritance of Man® website (www.OMIM.org) was used to search phenotype association #601367 (ischemic stroke) for associated genes and references. From these sources, a list of 75 gene names was derived, of which 71 could be investigated in Partek® Genomics Suite® (Table 1). The list included genes implicated in any stroke GWAS study, including non-replicated genes such as NINJ2 and WNK1. Additionally, selected genes implicated as vascular risk factors (VRF) specifically for stroke were included in the analyses (*n* = 71 genes) (Table 1). Due to the dynamic nature of this field of study, this could not be an exhaustive list of risk-associated genes. Thus, forkhead box F2 (FOXF2) and other recently identified loci were not included in the original analyses, but rather were investigated at gene- and transcript-levels after all other analyses were completed. Further, VRF genes were selected based upon a published association with stroke and, here again, we did not set a goal of compiling a comprehensive list of VRF genes.

**Table 1:** List of 71 ischemic stroke (IS) and IS sub-type associated risk genes derived from previously published studies.

**Gene symbol/References**	**Gene name**	**Risk association**
ABO (1, 24, 25)	Alpha 1–3-galactosyl-transferase	IS-subtype LVD
ACE (10, 26)	Angiotensin I converting enzyme	IS, LVD, SVD
ADD1 (27, 28)	Adducin 1	IS
AIM1 (29)	Absent in melanoma 1	IS
ALDH2 (24)	Aldehyde dehydrogenase 2 family (Mitochondrial)	IS-subtype SVD
ALOX5AP (10)	Arachidonate 5-lipoxygenase activating protein	IS—all subtypes
ANGPT1 (10)	Angiopoietin-1	IS—all subtypes
APOE/APOC1 (10, 26)	Apolipoprotein E/Apolipoprotein C1	IS, LVD, SVD
CDKN2A/CDKN2B/ (1, 10, 24, 26)	Cyclin dependent kinase inhibitor 2A/Cyclin dependent kinase inhibitor B/	IS—all subtypes
CRP (10)	C-reactive protein	IS-subtype LVD
CYP4A11 (10)	Cytochrome P450 family 4 subfamily A Member 11	IS
CYP4F2 (10)	Cytochrome p450 family 4 subfamily F Member 2	LVD, SVD
CYP11B2 (10)	Cytochrome P450 family 11 subfamily B Member 2	LVD, SVD
DDAH1 (10)	Dimethylarginine dimethylamino-hydrolase 1	IS—all subtypes
EPHX2 (9, 10)	Epoxide hydrolase 2	IS
F2 (prothrombin) (10, 26)	Coagulation factor II, Thrombin	IS-subtype LVD
F5 (factor V Leiden) (10, 26)	Coagulation factor V	IS—all subtypes
F7(26)	Coagulation factor VII	IS
F13A1/F13B (FXIII) (26)	Coagulation factor XIII	IS
FGA/FGB (10)	Fibrinogen alpha chain/Fibrinogen beta chain	IS—all subtypes
GP1BA (10, 26)	Glycoprotein Ib platelet alpha subunit	IS-subtype LVD
HABP2 (30)	Hyaluronan binding protein 2	IS
HDAC9 (24, 25)	Histone deacetylase 9	IS-subtype LVD
IL1A (31)	Interleukin 1 alpha	IS
IL6 (10)	Interleukin 6	IS—all subtypes
IMPA2 (29)	Inositol monophosphatase 2	IS
ITGA2B (GPIIb) (10, 26)	Integrin subunit alpha 2b	IS
ITGB3 (GPIIIa) (10, 26)	Integrin subunit beta 3	IS—all subtypes
LDLR/SMARCA4(1)	Low density lipoprotein receptor/SW1/SNF Related, Matrix Associated, Actin dependent regulator of chromatin, Subfamily A, Member 4	IS
LPAL2/SLC22A3/LPA (APOA) (1, 32)	Lipoprotein(A) Like 2, Pseudogene/Solute Carrier Family 22 Member 3/ Lipoprotein(A)	IS
LPL (26)	Lipoprotein lipase	IS-subtype SVD
LTA (33, 34)	Lymphotoxin alpha	IS
LTC4S (10) *annotated as MAML1 (same genomic location) in PGS*	Leukotriene C4 synthase	IS-subtype CE
MMP12 (25)	Matrix metallopeptidase 12	IS-subtype LVD
MTHFR (10, 26)	Methylenetetrahydrofolate reductase	IS—all subtypes
NAA25 (C12orf30) (25)	N(Alpha)-Acetyltransferase 25, NatB Auxiliary Subunit	IS
NINJ2 (10, 24–26, 35)	Ninjurin 2	CE, LVD
NOS1 (36)	Nitric oxide synthase 1	IS
NOS3 (eNOS) (10, 26)	Nitric oxide synthase 3	CE, SVD
NPY (10)	Neuropeptide Y	IS
PCSK9 (10)	Proprotein Convertase Subtilisin/Kexin Type 9	CE, LVD
PDE4D (10, 26)	Phosphodiesterase 4D	CE, LVD
PDGFC (37)	Platelet Derived Growth Factor C	IS (significant lncRNA nearby)
PITX2 (10, 24–26)	Paired like homeodomain 2	CE
PLPP3 (PPAP2B) (1)	Phospholipid phosphatase 3 (Phosphatidic acid phosphatase 2b)	IS
PON1 (10)	Paroxonase-1	IS—all subtypes
PRKCH (38)	Protein kinase C eta	IS
PTGIS (37)	Prostaglandin I2 Synthase	IS (significant lncRNA nearby)
RAI1-PEMT-**RASD1** (1)	Retinoic Acid Induced 1 Phosphatidylethanolamine N-Methyl-transferase **Ras related dexamethasone induced 1**	IS
SERPINE1 (PAI-1) (10, 26)	Serpin family E member 1 (Plasminogen Activator Inhibitor, Type I)	CE, LVD
SGK1 (10)	Serum/Glucocorticoid regulated kinase 1	CE, LVD
SH2B3 (1, 10, 24)	SH2B Adaptor Protein 3	IS, LVD
SLC4A1/APOL2 (39)	Solute carrier family 4 member 1 (Diego blood group)/Apolipoprotein L2	IS
SORT1 (1)	Sortilin 1	IS
SUPT3H/CDC5L (24, 25, 39)	SPT3 Homolog, SAGA And STAGA complex Component/Cell division cycle 5 like	IS-subtype LVD
TNF (40, 41)	Tumor necrosis factor alpha	IS
WNK1 (35, 42)	WNK lysine deficient protein kinase 1	IS
ZC3HC1 (1)	Zinc Finger C3HC-Type containing 1	IS
ZFHX3 (10, 24–26)	Zinc finger homeobox 3	CE, LVD
ZPR1 (ZNF259) (1)	ZPR1 zinc finger (Zinc finger protein 259)	IS

*The single gene not significantly differentially expressed in this study is bolded in the table below. CE, cardioembolism IS; LVD, large vessel disease IS; SVD, small vessel disease/ lacunar IS*.

### Study Subjects

**Figure 1:**
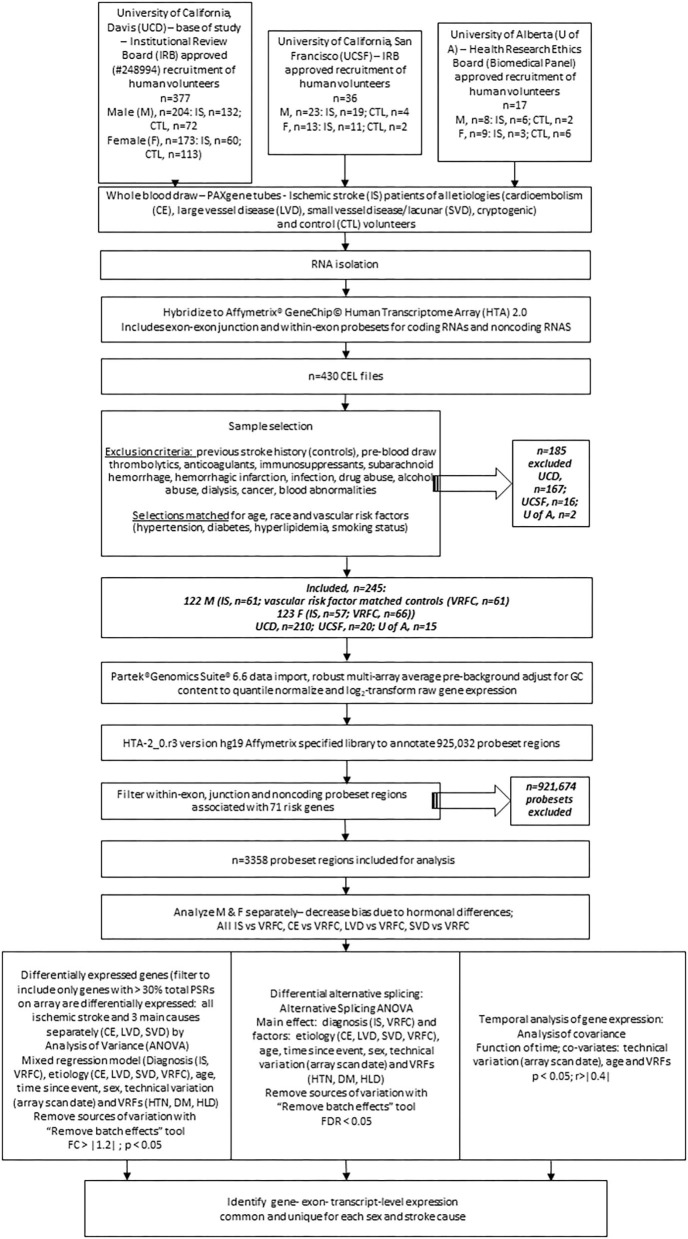
Experimental flowchart showing steps followed for analysis of gene expression in the peripheral blood of human ischemic stroke patients and control subjects recruited at three medical centers. M, male; F, female; IRB, internal review board; UCD, University of California, Davis; UCSF, University of California; San Francisco; U of A, University of Alberta; nc, non-coding; VRF, vascular risk factor; HTN, hypertension; DM, diabetes mellitus; HLD, hyperlipidemia; IS, ischemic stroke; VRFC, vascular risk factor matched control; RMA, robust multi-array average; DEG, differentially expressed gene; PSR, probeset region; CE, cardioembolism; LVD, large vessel disease; SVD, small vessel disease; DAS, differential alternative splicing.

Stroke and control subjects were recruited from 2005 through 2013 in medical centers at the Universities of California (UCs) at Davis and San Francisco and at the University of Alberta, Canada (see experimental flow chart, Figure 1). The studies involving human participants were reviewed and approved by the UC Davis Institutional Review Board (IRB) as the base of the study (IRB#248994), with additional site approvals by the UC San Francisco IRB and the University of Alberta Health Research Ethics Board (Biomedical Panel). The studies adhere to all federal and state regulations related to the protection of human research subjects, including The Common Rule, the principles of The Belmont Report, and Institutional policies and procedures. Written informed consent was obtained from all participants or their proxy. Board-certified neurologists diagnosed stroke as previously described (37).

Samples were matched for age, race, and vascular risk factors, including hypertension, diabetes mellitus, hyperlipidemia, and smoking status. Stroke patient samples were additionally matched for blood draw time since stroke onset. Exclusion criteria included history of previous stroke in controls, pre-blood draw thrombolytics, anticoagulants or immunosuppressants, subarachnoid or intracerebral hemorrhage, hemorrhagic transformation, infection, drug abuse, dialysis, cancer, and blood abnormalities.

### Blood Collection and RNA Isolation

Blood was collected via venipuncture into PAXgene tubes and RNA isolated as previously described (37). PAXgene tubes for blood collection are pre-filled with a solution that lyses the cells and stabilizes the RNA, which prevents degradation during storage.

### Array Design and Array Hybridization

Hybridization to Affymetrix GeneChip® Human Transcriptome 2.0 arrays (Affymetrix, Santa Clara, CA) was performed as described previously (37). The HTA 2.0 array includes exon-exon junction (JUC) and within-exon (PSR) probesets for coding and non-coding RNAs. Control and stroke samples were randomly allocated to microarray batches and scan date was a covariate in statistical analyses.

### Data Normalization and Statistical Analysis

For this exploratory investigation, probes were imported using Partek® Genomics Suite® software version 6.6 (Partek Inc., St. Louis, MO, USA). Robust multi-array average with pre-background adjustment for GC content was used to quantile normalize and log_2_-transform raw gene expression. After import, 925,032 probeset regions (PSRs) were annotated using Affymetrix specified library files HTA-2_0.r3 version hg19 and then filtered to include only 3,358 within-exon, junction and non-coding PSRs associated with the 71 previously identified risk genes (Table 1).

Exploratory analyses of gene- and probeset-specific (including probesets within exons and probesets spanning exon-exon junctions) differential expression were performed using a mixed regression model in Partek® Genomics Suite®. Factors in the model included: diagnosis [IS, vascular risk factor matched control (VRFC)], cause of stroke (CE, LVD, small vessel disease/lacunar (SVD), and VRFC), age, time since event, sex, technical variation (array scan date), and VRFs (hypertension, diabetes mellitus and hyperlipidemia). Although samples were collected from three different medical centers, we did not consider this as a factor in our model because the majority were collected from UC Davis (88%) with very few samples from UC San Francisco (8%) and University of Alberta (4%). However, after filtering the samples on the genes of interest list, we determined that there were no outliers from the University of California, San Francisco or the University of Alberta.

Analyses were performed separately on male and females to decrease bias related to hormonal differences and because sexual dimorphism exists in stroke (43, 44). Gene-, exon- and transcript-level expression common to both sexes and unique for each were identified. Differential expression was considered significant with absolute fold change (FC) > 1.2 and *p* < 0.05. Additionally, differentially expressed genes were filtered to include only significant genes for which more than 30% of the total number of probesets on the array for any particular gene were differentially expressed.

Genes with differential alternative splicing (DAS) were investigated using diagnosis as the main effect in an AS analysis of variance as per Partek algorithm that included subject ID and the same factors used in the mixed regression model. Genes displaying DAS with false discovery rate *p* (FDR) <0.3 were considered significant. A more relaxed FDR *p*-value was chosen due to the stringency of the Partek software Splicing ANCOVA Model (45–47) algorithm that was used to contrast Ischemic Stroke Diagnosis and Vascular Risk Factor Control groups, i.e.,:

Y_ijklmnop_ = μ + ScanDate_i_ + Diagnosis_j_ + Age + Time Since Stroke Symptom Onset + Hypertension_k_ + Diabetes_l_ + Hypercholesterolemia_m_ + MarkerID_n_ + SampleID (Scan Date ^*^ Diagnosis ^*^ Hypertension ^*^ Diabetes ^*^ Hypercholesterolemia)_ijklmo_ + Diagnosis ^*^ MarkerID_jn_ + ϵ_ijklmnop_

Where:

- Y_ijklmnop_ represents the p^th^ observation on the i^th^ Scan Date j^th^ Diagnosis k^th^ Hypertension l^th^ Diabetes m^th^ Hypercholesterolemia n^th^ Marker ID o^th^ Sample ID
- μ is the common effect for the whole experiment.
- ε_ijklmnop_ represents the random error present in the p^th^ observation on the i^th^ Scan Date j^th^ Diagnosis k^th^ Hypertension l^th^ Diabetes m^th^ Hypercholesterolemia n^th^ Marker ID o^th^ Sample ID. The errors ε_ijklmnop_ are assumed to be normally and independently distributed with mean 0 and standard deviation δ for all measurements.
- Marker ID_n_ is exon-to-exon effect (alt-splicing independent to group). This term also accounts for the fact that not all exons of a gene hybridize to the corresponding probe sets (MarkerID) with the same efficiency.
- Diagnosis ^*^ Marker ID_jn_ represent whether an exon expresses differently in different level of the specified Alternative Splice Factor(s).
- Sample ID (Scan Date ^*^ Diagnosis ^*^ Hypertension ^*^ Diabetes ^*^ Hypercholesterolemia)_ijklmo_ is a sample-to-sample effect. Sample ID and Scan Date are random effects.

Determination of differential gene expression between the three main causes of ischemic stroke, CE, LVD, and SVD, was made by performing sex-specific analysis of variance (ANOVA) on each subtype vs. VRFC. An analysis of covariance (ANCOVA) was performed for each sex as a function of time, with technical variation (array scan date), age and VRFs as co-variates. A list (*p* < 0.05) was derived and those with a partial correlation r > |0.4| were considered most significant (47).

## Results

### Participant Characteristics

There were 377 samples collected from volunteers recruited at UC Davis (UCD) Medical Center (M, *n* = 132 IS patients, *n* = 72 control subjects (CTLs); F, *n* = 60 IS patients, *n* = 113 CTLs). Additionally, 36 samples were collected from volunteers recruited at UC San Francisco (UCSF; M, *n* = 19 IS patients, *n* = 4 CTLs; F, *n* = 11 IS patients, *n* = 2 CTLs) and 17 samples were collected from volunteers recruited at University of Alberta (U of A; M, *n* = 8 IS patients, *n* = 2 CTLs; F, *n* = 9 IS patients, *n* = 6 CTLs). Our exclusion criteria eliminated 185 (UCD, *n* = 97 IS patients, *n* = 70 CTLs; UCSF, *n* = 14 IS patients, *n* = 2 CTLs; U of A, *n* = 2 IS patients) of the 430 total samples (Figure 1). Of the 245 remaining samples, 122 were male (IS, *n* = 61; VRFC, *n* = 61) and 123 were female (IS, *n* = 57; VRFC, *n* = 66). Arrays for all groups were run and scanned between February 15 and September 5, 2015. Patient and clinical characteristics are presented in Table 2. There were no statistical differences between male (M) and female (F) groups. Average age (in years) for M-VRFC was 58.71 ± 14.2 years; for F-VRFC−61.5 ± 12.2; for M-IS−61.4 ± 12.1; and for F-IS−66.00 ± 13.4. The number of subjects with hypertension were not statistically different for F-VRFC (44%) vs. F-IS (64%) or for M-VRFC (70%) vs. M-IS (75%). Similarly, subjects were matched for diabetic status (15% in F-VRFC, 28% in F-IS, 23% in M-VRFC, 30% in M-IS), hyperlipidemia (38% in F-VRFC, 41% in F-IS, 48% in M-VRFC, 40% in M-IS), and smoking (16% in F-VRFC, 23% in F-IS, 14% in M-VRFC, 19% in M-IS). Patients with cryptogenic strokes were included only in all IS patients vs. VRFC analyses. The average time since stroke did not differ statistically in male and female patients or between different stroke causes (Table 2). Time of blood draw after symptom onset ranged from 4.42 (h) to 134.25 h in M stroke patients and from 6 to 156 h in F patients.

**Table 2:** Characteristics of male and female ischemic stroke patients and control subjects.

**Male** **(*n* = 122)**	**Vascular risk** **factor matched** **control (*n* = 61)**	**Ischemic stroke** **(*n* = 61)**	**Cardio-embolic** **(*n* = 15)**	**Large vessel** **disease (*n* = 8)**	**Small vessel disease/** **Lacunar** ***(n* = 17)**	**Crypto-genic** **(*n* = 21)**
Age (mean, years ± SD)	58.71 ± 14.17	61.39 ± 12.09	58.68 ± 11.19	67.69 ± 8.58	59.59 ± 11.69	62.40 ± 13.79
Time Since Event (mean, hours ± SD)	not applicable	49.01 ± 27.17	42.58 ± 26.36	63.49 ± 24.43	52.76 ± 32.42	45.06 ± 23.13
Race, (% white)	55.74%	55.74%	53.33%	90.48%	37.50%	23.53%
**Race (*****n*****)**						
Asian	11	7	4	1	2	0
Black or African American	4	11	1	3	6	1
Latino	7	5	1	0	4	0
Mixed race	5	3	1	0	1	1
Native Hawaiian or Other pacific islander	0	1	0		0	0
White	34	34	8	3	4	19
Hypertension (n)	27	39	11	6	11	11
Diabetes (*n*)	9	17	3	1	7	6
Hyper-lipidemia (*n*)	23	25	3	3	10	9
Smoker (*n*)	10	14	6	2	2	4
**Female** **(*****n*** **=** **123)**	**Vascular risk** **factor matched** **control (*****n=*** **66)**	**Ischemic stroke** **(*****n=*** **57)**	**Cardio-embolic** **(*****n*** **=** **15)**	**Large vessel** **disease (*****n*** **=** **8)**	**Small vessel disease/** **Lacunar (*****n*** **=** **17)**	**Crypto-genic** **(*****n*** **=** **21)**
Age (mean, years ± SD)	61.51 ± 12.23	65.24 ± 13.4	65.98 ± 13.36	72.83 ± 9.57	60.34 ± 14.59	66.58 ± 12.76
Time Since Event (mean, hours ± SD)	not applicable	56.71 ± 34.58	55.32 ± 33.21	61.84 ± 31.55	64.43 ± 41.75	49.86 ± 30.57
Race (% white)	41.18%	63.41%	60.61%	76.92%	80.95%	66.67%
**Race (*****n*****)**						
Asian	4	2	0	0	2	0
Black or African American	7	8	4	1	0	3
Latino	8	2	1	0	0	1
Mixed race	6	5	1	2	0	2
Native hawaiian or Other pacific islander	0	2	0	1	0	1
White	40	38	9	4	15	14
Hypertension (*n*)	46	43	7	5	16	15
Diabetes (*n*)	15	17	3	3	6	5
Hyper-lipidemia (*n*)	32	23	4	3	7	9
Smoker (*n*)	9	11	0	0	3	8

### Differential Gene-Level Expression of Risk Genes in Stroke Patients Compared to Matched Control Subjects

#### Differential Gene Expression of Risk Genes Differed Between the Sexes and Between the Three Main Causes of IS

**Figure 2:**
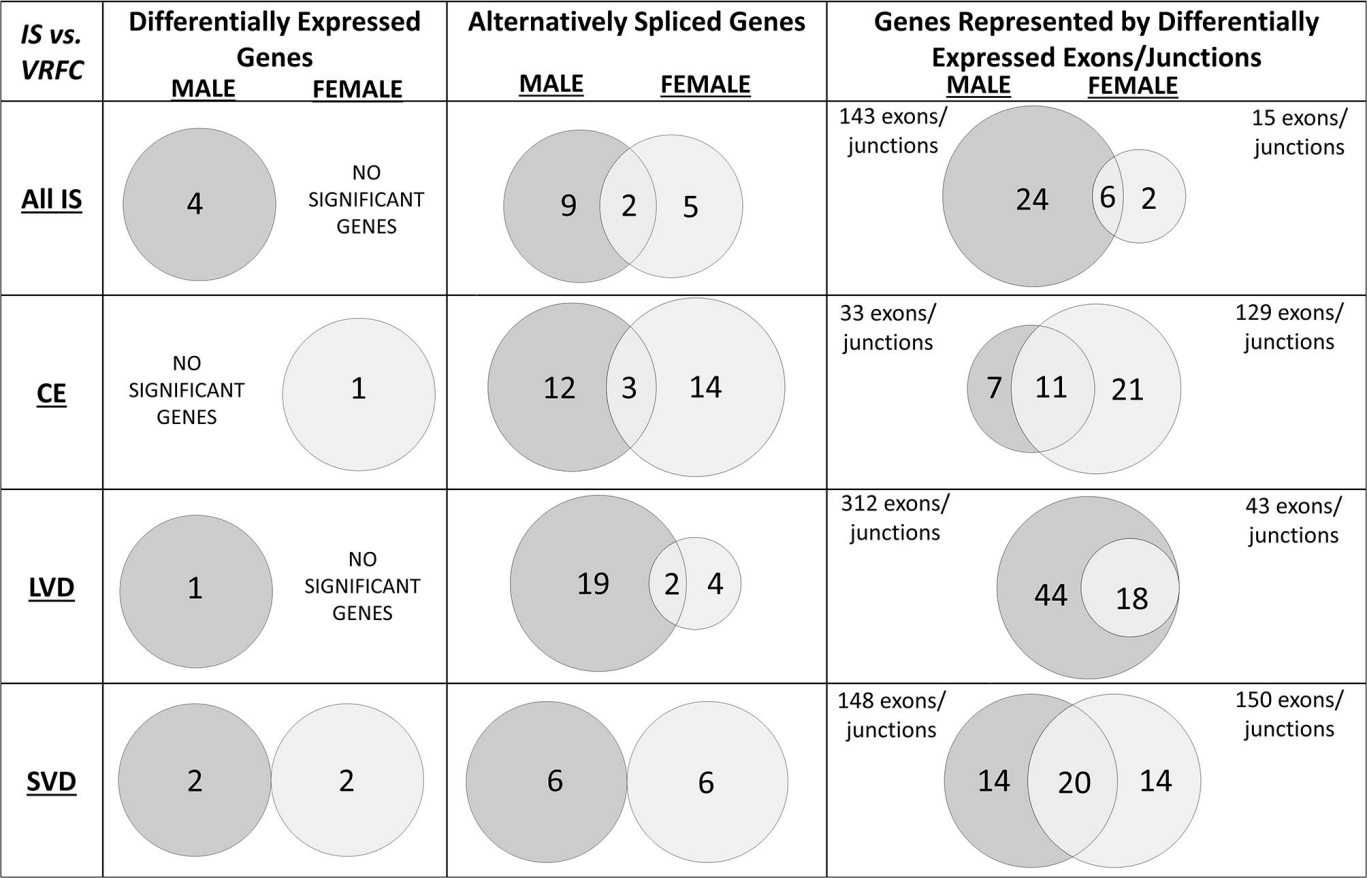
Venn diagrams of significant differentially expressed male and female genes for all ischemic stroke patients compared to control subjects and for each of the three main causes of ischemic stroke: cardioembolic (CE), large vessel disease (LVD), small vessel disease/lacunar (SVD). Differences in mRNA expression were assessed at the gene level (median of all probesets for each gene), alternative splicing level, and exon/junction level. Associated genes are listed in Table 3. Actual statistical significance (*p* < 0.05) and fold change (FC > |1.2|) values are presented in Table 4 (Differentially expressed genes), Table 5 (Alternatively spliced genes), and Supplementary Tables 1–3 (Genes represented by differentially expressed exons/junctions). See methods section for statistical analysis protocol. CE, cardioembolic IS; SVD, small vessel disease/lacunar IS; LVD, large vessel disease IS.

Four genes were significantly differentially expressed (DE) between all IS male patients and VRFC males, including: aldehyde dehydrogenase 2 family (mitochondrial) (ALDH2), arachidonate 5-lipoxygenase activating protein (ALOX5AP), coagulation factor XIII A chain (F13A1), and inositol monophosphatase 2 (IMPA2) (Figure 2, Table 3). All had decreased (FC < −1.2) expression levels (Table 4). No significant genes were found when all female IS patients were compared to female VRFCs (Figure 2, Tables 3, 4).

**Table 3:** Significant differentially expressed genes represented in Venn Diagrams of Figure 2.

**IS vs** **VRFC**	**Differentially expressed genes**		**Alternatively spliced genes**			**Genes represented by differentially expressed** **exons/Junctions**		
	**M**	**F**	**M**	**Common M-F**	**F**	**M**	**Common M-F**	**F**
All IS	ALDH2, ALOX5AP, F13A1, IMPA2	No sig-nificant genes	ADD1, ITGB3, LPA, LPAL2, NINJ2, PTGIS, SORT1, WNK1, ZFHX3	GP1BA, ITGA2B	F5, MTHFR, PON1, PRKCH, SMARCA4	ADD1, ALDH2, ALOX5AP, EPHX2, F13A1, F7, GP1BA, IMPA2, LDL4, LPL, LTA, LTC4S, NINJ2, NOS1, PCSK9, PDE4D, PEMT, SGKA, SH2B3, SUPT3H, WNK1, ZC3HC1, ZFHX3, ZPR1	ACE, HDAC9, ITGB3, MTHFR, SMARCA4, SORT1	CDC5L, PRKCH
CE	No sig-nificant genes	ITGB3	ALOX5AP, APOL2, CDKN2A, GP1BA, ITGA2B, LPA, NINJ2, PCSK9, PDGFC, SH2B3, WNK1, ZFHX3	ADD1, ITGB3, LDLR	ALDH2, EPHX2, F5, HDAC9, MTHFR, NAA25, PDE4D, PEMT, PON3, PRKCH, SMARCA4, SUPT3H, ZC3HC1, ZPR1	ABO, APOL2, CDKN2A, CYP4A11, EPHX2, F5, TNF	ADD1, ALDH2, F7, FGB, HDAC9, IMPA2, MTHFR, NOS3, PDE4D, PON1, ZPR1	ACE, AIM1, ANGPT1, CYP4F2, DDAH1, GP1BA, ITGA2B, ITGB3, LDLR, LPA, LPL, NAA25, PDGFC, PEMT, PRKCH, SGK1, SH2B3, SMARCA4, SORT1, WNK1
LVD	ADD1	No sig-nificant genes	ADD1, ALDH2, CYP4A11, DDAH1, EPHX2, F2, FGB, HDAC9, IMPA2, LPA, MTHFR, NOS1, NPY, PCSK9, PDGFC, PLPP3, PON3, SGK1, SMARCA4	F13B, IL1A	F13A1, F5, LDLR, PDE4D	ABO, ALOX5AP, ANGPT1, APOC1, APOE, CDC5L, CDKN2A, CRP, CYP11B2, CYP4A11, DDAH1, F13G, F2, F5, F7, FGB, HABP2, IL1A, IMPA2, ITGA2B, LDL4, LPA, LPL, LTA, LTC4S, MMP12, NINJ2, NOS1, NPY, PCSK9, PEMT, PITX2, PLPP3, PON1, PTGIS, RAI1, SERPINE1, SGK1, SH2B3, SMARCA4, SORT1, SUPT3H, ZC3HC1, ZPR1	ACE, ADD1, AIM1, ALDH2, APOL2, CDKN2B, EPHX2, HDAC9, ITGB3, LPAL2, MTHFR, NAA25, NOS3, PDE4D, PDGFC, PRKCH, WNK1, ZFHX3	
SVD	F13A1, IMPA2	WNK1, WNK1-lncRNA	CYP4F2, HDAC9, LPAL2, NINJ2, SORT1, WNK1	No common significant genes	ALDH2, F13A1, MTHFR, PON1, SMARCA4, ZFHX3	AIM1, ALOX5AP, CDC5L, F13A1, F13B, HDAC9, IMPA2, ITGA2B, LPAL2, LPL, LTC4S, MTHFR, PDGFC, ZFHX3	ACE, ADD1, ALDH2, APOL2, CYP4F2, DDAH1, GP1BA, ITGB3, LDLR, LPA, NINJ2, NOS3, PCSK9, PDE4D, PEMT, SGK1, SLC4A1, SMARCA4, SORT1, WNK1	CYP4A11, EPHX2, F2, F7, FGA, NAA25, NOS1, PITX2, PLPP3, RAI1, SH2B3, SLC22A3, ZC3HC1, ZPR1

*Significance: fold change (FC) > |1.2|, p < 0.05, false discovery rate p (FDR) < 0.3 (for alternative splicing); IS, ischemic stroke; VRFC, vascular risk factor matched controls; CE, cardioembolism IS; LVD, large vessel disease IS; SVD, small vessel disease/lacunar IS; M, male; F, female. Exact fold change and p-values are presented in Tables 4, 6–8; Supplementary Tables 1–3*.

**Table 4:** Significant differentially expressed genes between stroke patients and matched controls in male and female cohorts.

**Sex**	**Gene symbol**	**Affymetrix transcript** **clusters**	**Stroke vs.** **VRFC**	***p*-value**	**Fold change**
Male	ADD1	TC04000039	LVD	4.42E-02	−2.03
	ALDH2	TC12003212	ALL IS	4.97E-02	−1.28
	ALOX5AP	TC13000100		1.49E-02	−1.49
	F13A1	TC06001246	ALL IS	2.38E-02	−1.45
			SVD	1.93E-03	−2.62
	IMPA2	TC18000060	ALL IS	1.81E-02	−1.50
			SVD	5.27E-03	−2.17
Female	ITGB3	TC17002878	CE	2.30E-02	−2.97
	WNK1	TC12000010	SVD	4.91E-03	1.79
	WNK1-non-coding	TC12002169		1.13E-02	1.63

*Significance: fold change (FC) > |1.2|; p < 0.05. Negative fold change indicates decreased expression. Positive fold change indicates increased expression. IS, ischemic stroke; VRFC, vascular risk factor matched controls; CE, Cardioembolism IS; LVD, Large Vessel Disease IS; SVD, Small Vessel Disease/Lacunar IS*.

#### Differential Gene Expression in Cardioembolism Cause of Ischemic Stroke

At the gene level, integrin subunit beta 3 (ITGB3) had significantly lower expression in female CE patients compared to female VRFC subjects whereas there were no differentially expressed genes in male CE patients (Figure 2, Tables 3, 4).

#### Differential Gene Expression in Large Vessel Disease Cause of Ischemic Stroke

Adducin 1 (ADD1) had significantly decreased expression in male LVD patients compared to VRFCs, whereas there were no differentially expressed genes in females (Figure 2, Tables 3, 4).

#### Differential Gene Expression in Small Vessel Disease/Lacunar Cause of Ischemic Stroke

F13A1 and IMPA2 had significantly decreased expression in male SVD patients compared to VRFCs (Figure 2, Tables 3, 4). In female SVD patients, WNK lysine deficient protein 1 (WNK1) and WNK1 mRNA-like long non-coding RNA (lncRNA; accession DQ925669) had significantly increased expression in SVD patients compared to VRFCs (Figure 2, Tables 3, 4).

### Differential Alternative Splicing in the Risk Genes in Ischemic Stroke Patients Compared to Matched Control Subjects

**Figure 3:**
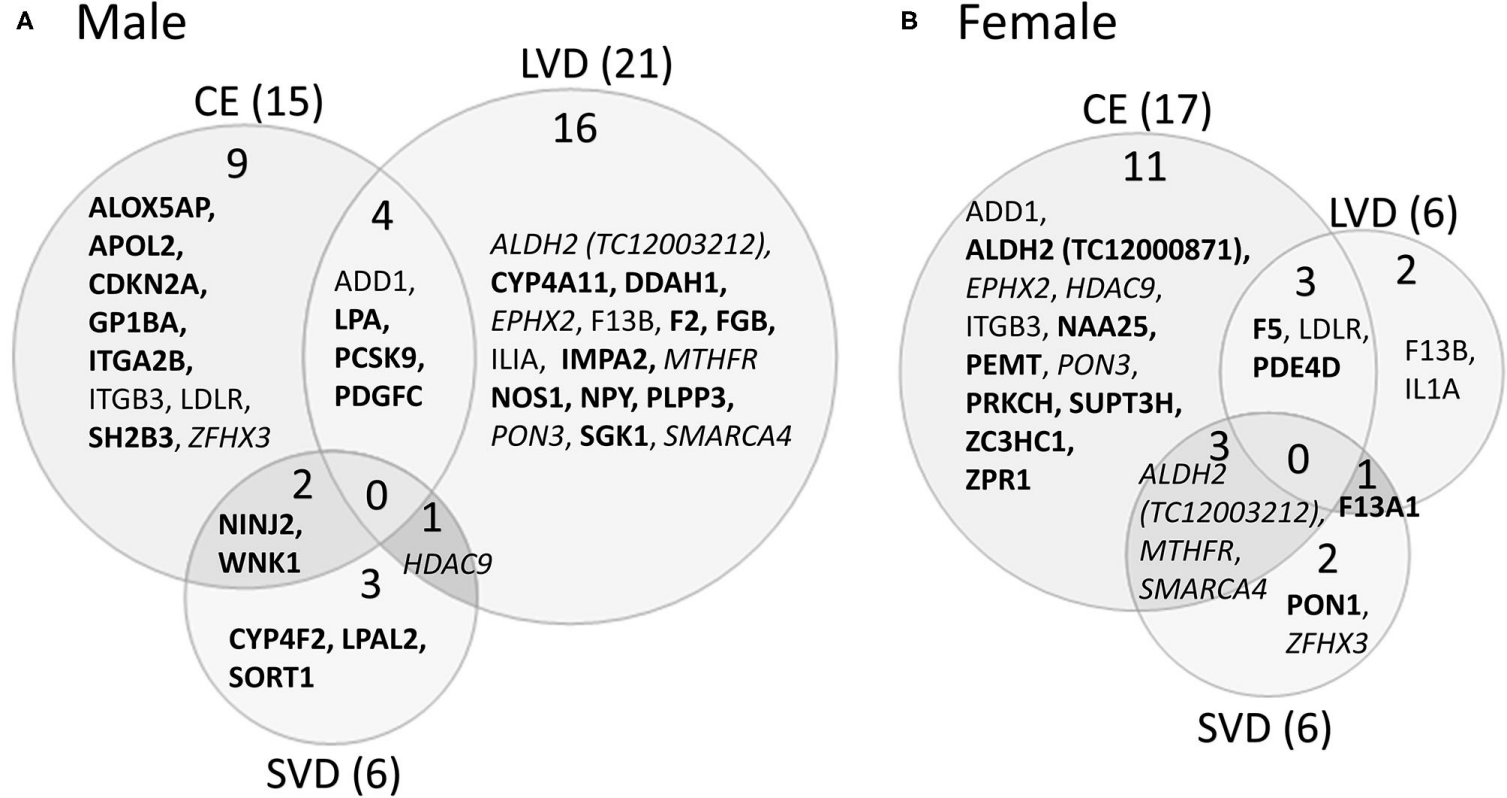
Venn diagram depicting alternatively spliced genes by cause of ischemic stroke in male **(A)** and female **(B)** stroke patients compared to vascular risk factor control subjects. These genes are derived from the middle column of Figure 2. Numbers in parentheses represent the number of ischemic stroke cause-specific genes for each sex. Bold font indicates sex specific genes. Italicized font indicates common alternatively spliced genes between sexes but within different causes of IS. Actual statistical significance values for the male cohort are presented in Table 5A and the female cohort in Table 5B. See methods section for statistical analysis protocol. CE, cardioembolic IS; SVD, small vessel disease/lacunar IS; LVD, large vessel disease IS.

A striking find related to AS is that most of the genes with predicted AS for each of the three main causes of stroke investigated are generally unique to each stroke cause (Figure 3, Tables 3, 5–7). This is emphasized by the fact that only two genes histone deacetylase 9 (HDAC9) and ALDH2, are predicted to have AS in all three causes of stroke (CE, LVD, and SVD) in either male or female patients (Figure 3, Tables 6, 7).

**Table 5:** Genes significant for alternative splicing (AS) in the (A) male and (B) female cohorts for all ischemic stroke (IS) and the three main causes of stroke.

**Gene** **symbol**	**Affymetrix transcript clusters**	**AS** **(Dx-All IS)** ***p*-value**	**AS** **(Dx-CE)** **FDR**	**AS** **(Dx-LVD)** **FDR**	**AS** **(Dx-SVD)** **FDR**
**A. GENES SIGNIFICANT FOR AS IN THE MALE COHORT**					
ADD1	TC04000039	2.81E-09	2.84E-05	2.62E-03	
*ALDH2*	*TC12003212*			*1.84E-05*	
**ALOX5AP**	**TC13000100**		**4.25E-03**		
**APOL2**	**TC22000702**		**2.38E-02**		
**CDKN2A**	**TC09000964**		**4.01E-02**		
**CYP4A11**	**TC01002625**			**6.98E-15**	
**CYP4F2**	**TC19001261**				**1.82E-06**
**DDAH1**	**TC01002829**			**2.79E-03**	
*EPHX2*	*TC08000208*			*4.81E-04*	
F13B	TC01003661			9.37E-03	
**F2**	**TC11000401**			**2.29E-02**	
**FGB**	**TC04000777**			**9.37E-03**	
**GP1BA**	**TC17000056**	**2.92E-08**	**2.28E-04**		
*HDAC9*	*TC07000116*			*1.09E-14*	*1.40E-02*
IL1A	TC02002218			1.33E-03	
**IMPA2**	**TC18000060**			**3.82E-04**	
**ITGA2B**	**TC17001581**	**4.03E-04**	**1.00E-09**		
ITGB3	TC17002878	2.40E-04	1.77E-08		
LDLR	TC19000191		1.43E-03		
**LPA**	**TC06002281**	**1.93E-04**	**2.15E-02**	**4.57E-03**	
**LPAL2**	**TC06002280**	**4.20E-05**			**4.51E-03**
*MTHFR*	*TC01002202*			*2.96E-03*	
**NINJ2**	**TC12001083**	**2.40E-08**	**2.01E-19**		**3.90E-02**
**NOS1**	**TC12002014**			**1.77E-02**	
**NPY**	**TC07000153**			**4.57E-03**	
**PCSK9**	**TC01000675**		**3.87E-04**	**1.77E-02**	
**PDGFC**	**TC04001673**		**6.64E-03**	**1.77E-02**	
**PLPP3**	**TC01002701**			**9.37E-03**	
*PON3*	*TC07003364*			*4.81E-04*	
PTGIS	TC20000927	2.65E-04			
**SGK1**	**TC06002126**			**2.17E-02**	
**SH2B3**	**TC12000868**		**8.39E-07**		
*SMARCA4*	*TC19000190*			*1.83E-12*	
**SORT1**	**TC01002954**	**3.66E-05**			**3.90E-02**
**WNK1**	**TC12000010**	**2.07E-05**	**1.02E-07**		**5.25E-04**
*ZFHX3*	*TC16001259*	*2.43E-03*	*2.17E-02*		
**B. GENES SIGNIFICANT FOR AS IN THE FEMALE COHORT**					
ADD1	TC04000039		7.88E-05		
**ALDH2**	**TC12000871**		**1.99E-02**		
*ALDH2*	*TC12003212*		*2.39E-02*		*2.36E-02*
*EPHX2*	*TC08000208*		*2.43E-21*		
**F13A1**	**TC06001246**			**3.36E-14**	**2.34E-05**
F13B	TC01003661			3.70E-04	
**F5**	**TC01003498**	**1.29E-05**	**1.04E-19**	**3.45E-15**	
**GP1BA**	**TC17000056**	**2.01E-09**			
*HDAC9*	*TC07000116*		*2.13E-05*		
IL1A	TC02002218			9.66E-03	
ITGA2B	TC17001581	5.97E-04			
ITGB3	TC17002878		2.61E-02		
LDLR	TC19000191		2.05E-04	2.42E-06	
*MTHFR*	*TC01002202*	*2.42E-03*	*4.42E-02*		*1.28E-03*
**NAA25**	**TC12001977**		**7.22E-07**		
**PDE4D**	**TC05001389**		**2.66E-15**	**2.16E-02**	
**PEMT**	**TC17001188**		**4.42E-03**		
**PON1**	**TC07003363**	**7.76E-06**			**1.28E-03**
*PON3*	*TC07003364*		*1.49E-07*		
**PRKCH**	**TC14000371**	**1.60E-13**	**6.84E-49**		
*SMARCA4*	*TC19000190*	*9.41E-08*	*5.74E-12*		*1.12E-03*
**SUPT3H**	**TC06001765**		**1.70E-06**		
**ZC3HC1**	**TC07001860**		**3.27E-02**		
*ZFHX3*	*TC16001259*				*9.37E-03*
**ZPR1**	**TC11002324**		**3.27E-02**		

*Significance: false discovery rate (FDR) <0.3. Bold font indicates sex-specific alternatively spliced genes. Italicized font indicates AS common between sexes, but which differ between stroke cause. CE, cardioembolism; LVD, large vessel disease; SVD, small vessel disease/lacunar*.

**Table 6:** Common exons/junctions and their representative genes in both sexes with opposite expression for three main causes of ischemic stroke.

**Stroke cause**	**Gene symbol**	**Affymetrix** **probe set ID**	**Male**		**Female**	
			***p*-value**	**Fold change**	***p*-value**	**Fold change**
*CE*	IMPA2	JUC18000387	3.93E-02	2.02	1.79E-02	−1.87
*LVD*	ALDH2	PSR12011484	3.39E-02	−1.60	4.39E-02	1.47
*SVD*	ADD1	JUC04000402	3.72E-02	−1.34	4.40E-02	1.30
		PSR04001025	3.11E-02	−1.42	3.97E-02	1.26
		PSR04001026	1.47E-02	−1.55	1.03E-02	1.42
		PSR04001030	3.76E-02	−1.56	3.06E-02	1.45
		PSR04001032	3.22E-02	−1.69	2.40E-02	1.42
		PSR04001038	9.87E-03	−1.46	3.65E-02	1.27
	NINJ2	PSR12014336	4.98E-02	−1.92	2.50E-02	1.71
	PCSK9	JUC01005610	5.31E-04	−2.75	3.18E-02	1.82
	PEMT	PSR17016158	4.63E-02	−1.25	3.04E-03	1.43
		PSR17016167	2.92E-02	−1.54	1.53E-02	1.48
	SMARCA4	JUC19001703	2.49E-02	−2.19	3.82E-02	1.68
	WNK1	JUC12000095	4.99E-03	−2.62	2.58E-02	1.99
		JUC12000121	2.41E-02	−2.43	1.02E-02	2.40
		JUC12000131	4.54E-02	−1.61	9.44E-04	1.84
		PSR12000128	3.31E-02	−1.71	1.72E-03	1.91
		PSR12000163	4.39E-02	−1.87	7.72E-03	2.01
		PSR12000165	2.92E-02	−1.66	6.95E-03	1.69
	WNK1-lncRNA	PSR12028413	4.65E-02	−1.39	8.09e-03	1.52

*Significant fold change and p-values are shown for common expression of exons and/or junctions. This table is derived from data presented in Supplementary Tables 2, 3. CE, cardioembolism; SVD, small vessel disease/lacunar; LVD, large vessel disease*.

**Table 7:** Common representative stroke cause-specific genes and associated exons/junctions in both sexes.

**A. STROKE-CAUSE SPECIFIC GENES COMMON IN BOTH SEXES REPRESENTED BY DIFFERENT EXONS AND JUNCTIONS**						
**Stroke cause**	**Gene symbol**	**Affymetrix** **probe set ID**	**Male**		**Female**	
			***p*****-value**	**Fold change**	***p*****-value**	**Fold change**
SVD	SLC4A1	JUC17011883	4.53E-02	−1.53		
		JUC17011885			5.44E-03	1.43
		JUC17011886			3.36E-02	1.66
		JUC17011893			1.30E-02	1.72
		PSR17021220			5.21E-04	2.26
LVD	CDKN2B	JUC09006956	2.97E-02	3.18		
		PSR09012783			4.8E-03	1.47
**B. GENES COMMON IN BOTH SEXES REPRESENTED BY DIFFERENT EXONS AND JUNCTIONS IN MALE LVD AND FEMALE SVD CAUSES OF**						
**ISCHEMIC STROKE**						
**Stroke cause-Sex**	**Gene symbol**	**Affymetrix** **probeset ID**	**Male**		**Female**	
			**p-value**	**Fold Change**	**p-value**	**Fold Change**
LVD-M; SVD-F	F2	PSR11005085	1.37E-02	2.35		
		PSR11005066			1.24E-02	−1.35
	NOS1	JUC12014770	8.87E-04	2.60		
		JUC12014780	5.92E-04	5.81		
		JUC12014783	2.97E-02	1.46		
		JUC12014789	2.45E-03	1.67		
		PSR12026409	3.90E-02	1.40		
		PSR12026415	5.83E-03	2.64		
		JUC12014768			7.77E-03	−1.73
		JUC12014792			1.93E-02	−1.42
	PITX2	JUC04010982	1.68E-02	1.74		
		PSR04020916			3.03E-02	−1.28
	PLPP3	PSR01042365	2.24E-02	2.10		
		PSR01042375			4.78E-02	−1.71
	RAI1	JUC17001559	1.60E-02	−2.30		
		JUC17001562	9.96E-03	3.86	3.54E-02	−1.72
		PSR17002766	3.32E-02	1.55		
	ZC3HC1	JUC07014252	4.89E-03	3.28		
		JUC07014254	3.18E-02	2.42		
		PSR07028796	5.44E-03	−1.76		
		PSR07028800	1.05E-02	1.61		
		PSR07028794			4.56E-02	1.49

*Significant fold change and p-values are shown for common cause-specific genes. Part A presents the common genes with differing associated exons and/or junctions for LVD and SVD stroke causes in both sexes. Part B presents the common genes for male LVD and female SVD and their associated exons and/or junctions. This table is derived from data presented in Supplementary Tables 2, 3. CE, cardioembolism; SVD, small vessel disease/lacunar; LVD, large vessel disease*.

#### Differential Alternative Splicing in All Causes of Ischemic Stroke

For all IS compared to VRF controls, there were eleven and seven genes significant for DAS (FDR < 0.3) in males and females, respectively (Figure 2, Table 5). Only two differentially spliced genes were common between both sexes: glycoprotein Ib platelet alpha subunit (GP1BA) and integrin subunit alpha 2b (ITGA2B) (Figure 2, Table 5). Genes significant for DAS in the male cohort (Table 5A) included: ADD1, integrin subunit beta 3 (ITGB3), lipoprotein A (LPA), lipoprotein A like 2, pseudogene (LPAL2), ninjurin 2 (NINJ2), prostaglandin I2 synthase (PTGIS), sortilin 1 (SORT1), WNK1, and zinc finger homeobox 3 (ZFHX3). Notably, PTGIS was previously reported to be in the vicinity of a lncRNA that was negatively correlated with time after stroke in male stroke patients (37), suggesting a role for non-coding RNA in differential gene expression. In the female cohort (Table 5B), genes significant for DAS included: coagulation factor V (F5)methylenetetrahydrofolate reductase (MTHFR), paroxinase-1 (PON1), protein kinase C eta (PRKCH), and SNF related matrix associated, actin dependent regulator of chromatin, subfamily A, member 4 (SMARCA4).

#### Differential Alternative Splicing in Cardioembolism Cause of Ischemic Stroke

In male CE patients compared to VRFCs, 15 genes were significant for DAS, including previously implicated GWAS CE risk genes: NINJ2, proprotein convertase subtilisin/Kexin type 9 (PCSK9) and ZFHX3 (Figure 3A, Table 5A). In female CE patients, 17 genes were significant for DAS, including ADD1, a gene previously reported in the vicinity of a long non-coding RNA that had increased expression in female IS patients compared to control subjects (37) (Figure 3B, Table 5B). Three genes, ADD1, ITGB3 and low-density lipoprotein receptor (LDLR), showed DAS in both sexes (Figure 3, Table 5).

#### Differential Alternative Splicing in Large Vessel Disease Cause of Ischemic Stroke

There were 21 genes significant for DAS in male LVD patients compared to VRFCs (Figure 3A, Table 5A). These included LVD risk genes: coagulation factor II, thrombin (F2) (10, 26), HDAC9 (24), and serum/glucocorticoid regulated kinase 1 (SGK1) (10). LPA was significant for DAS in males for both LVD and CE etiologies and had previously been reported in the vicinity of linc-SLC22A2, a lncRNA with increased differential expression in male IS patients compared to control subjects (37). In contrast, female LVD patients had only six genes significant for DAS (Figure 3B, Table 5B): F13A1, coagulation factor XIII B chain (F13B), F5, interleukin 1 alpha (IL1A), MTHFR and phosphodiesterase 4D (PDE4D), also alternatively spliced in CE patients and previously identified as a CE/LVD risk gene (10, 26). Two genes, F13B and IL1A, were significant for DAS in both sexes (Figure 3, Table 5).

#### Differential Alternative Splicing in Small Vessel Disease/Lacunar Cause of Ischemic Stroke

There were six genes significant for DAS in SVD for each of the sexes (Figure 3, Table 5). There were no common genes significant for DAS in male and female SVD patients (Figure 3, Table 5). In male SVD patients (Figure 3A, Table 5A), alternatively spliced genes included: cytochrome P450 family 4, subfamily F, member 2 (CYP4F2), a previously GWAS identified SVD risk gene (10) as well as HDAC9, LPAL2, NINJ2, SORT1, and WNK1. In females, genes significant for DAS included: ALDH2, a SVD risk gene (24), F13A1, MTHFR, PON1, SMARCA4, and ZFHX3 (Figure 3B, Table 5B).

### Differential Exon/Junction Usage in the Risk Genes of Ischemic Stroke Patients Compared to Matched Control Subjects

Interestingly, of the 71 risk genes that were investigated in this study, 70 (Supplementary Tables 1–3), had significant differential exon/junction usage, yet only four genes were common between sexes in all three main causes of stroke: ADD1, ALDH2, nitric oxide synthase 3 (NOS3) and PDE4D (Supplementary Tables 2, 3), indicating that differential exon and junction expression is sex- and stroke cause-specific. Of the 70 genes with significant differential exon/junction expression, 21 were specific to the male cohort (Supplementary Table 2) and two were female-specific: fibrinogen alpha chain (FGA) and solute carrier family 22 member 3 (SLC22A3) (Supplementary Table 3). Within the specific stroke causes, only nine genes were represented by 23 exons and junctions that were common between the sexes (Supplementary Tables 1–3), yet only one, ITGB3, showed exons/junctions with similar expression patterns in both sexes (Supplementary Tables 2, 3).

#### Differential Exon/Junction Usage in All Causes of Ischemic Stroke

Comparing all IS patients to VRFCs, there were 143 differentially expressed exons and junctions from 30 genes in males (Figure 2, Table 3, Supplementary Table 1) and 15 differentially expressed exons/junctions from eight genes in females (Figure 2, Table 3, Supplementary Table 1). The majority (88.11%) of differentially expressed exons/junctions in males decreased expression, whereas only about half (46.67%) decreased expression in females. Although there were six genes common to both sexes (angiotensin I converting enzyme (ACE), HDAC9, ITGB3, MTHFR, SMARCA4, and SORT1), these were represented by different exons/junctions (Figure 2, Table 6).

#### Differential Exon/Junction Usage in Cardioembolism Cause of Ischemic Stroke

**Figure 4:**
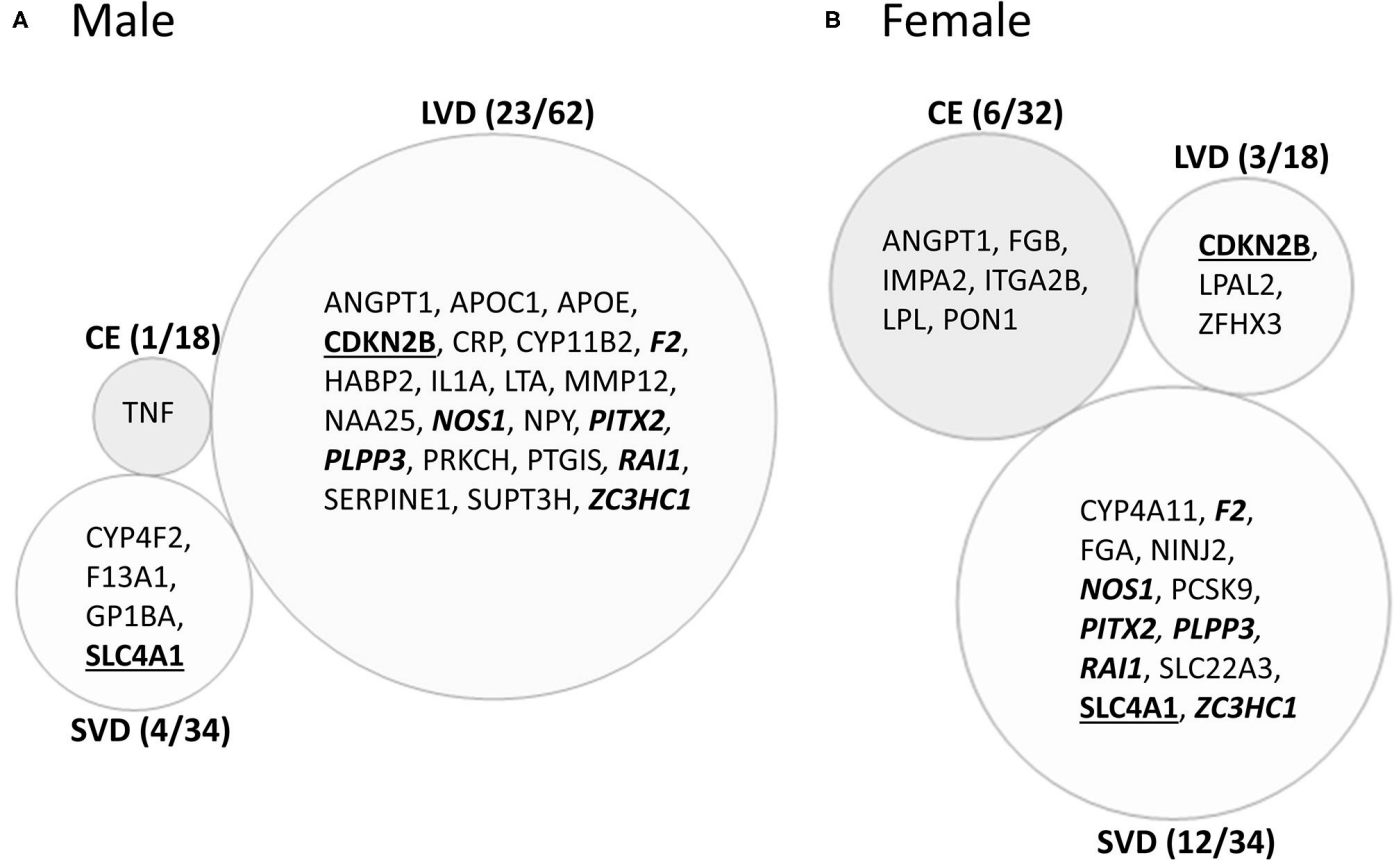
Venn diagram of ischemic stroke (IS) cause-specific genes with differentially expressed exons/junctions in ischemic stroke vs. vascular risk factor control subjects in the male **(A)** and in female **(B)** cohorts. These genes are derived from the far-right column of Figure 2. Numbers in parentheses represent the number of subtype-specific genes per number of total genes differentially expressed within each stroke subtype. Bold underlined gene symbols indicate common genes between male and female cohorts within the same stroke cause. Bold italicized genes are common between male LVD and female SVD. Actual statistical significance (*p* < 0.05) and fold change (FC>|1.2|) values are presented in Tables 6, 7 and Supplementary Tables 2, 3. See methods section for statistical analysis protocol. CE, cardioembolic IS; SVD, small vessel disease/lacunar IS; LVD, large vessel disease IS.

Male CE patients had 33 significant differentially expressed exons and junctions from 18 genes (Figure 2, Supplementary Table 2) with only one of those 18 genes, TNF, unique for CE (Figure 4A). Female CE patients had 129 differentially expressed exons/junctions representing 32 genes (Figure 2, Supplementary Table 3), with six of the 32 genes unique for CE: ANGPT1, fibrinogen beta chain (FGB), IMPA2, ITGA2B, lipoprotein lipase (LPL), and PON1 (Figure 4B). There were 11 genes common to males and females, including the CE associated risk gene, PDE4D (10, 26) (Figure 4, Supplementary Tables 2, 3). One junction, JUC18000387, in IMPA2 was commonly but inversely expressed in male and female patients (Table 6).

#### Differential Exon/Junction Usage in Large Vessel Disease Cause of Ischemic Stroke

In male LVD patients compared to VRFCs, 312 differentially expressed exons/junctions represented 62 genes (Figure 2, Table 3, Supplementary Table 2), of which 23 were expressed only in LVD (Figure 4A, Supplementary Table 2) and 12 were specific to the male cohort. Seven genes that are implicated in LVD risk and that had differentially expressed exons/junctions were specific to the male LVD cohort: Apolipoproteins C1 and E (APOC1/APOE) (10, 26), C-reactive protein (CRP) (10, 48), F2 (10, 26), matrix metallopeptidase 12 (MMP12) (25), serpine family E member 1 [SERPINE (PAI-1)] (10, 26), and SPT3 homolog, SAGA and STAGA complex component (SUPT3H) (24, 39, 49) (Figure 4A, Supplementary Table 2). In female LVD patients, 43 differentially expressed exons/junctions represented 18 genes (Figure 2, Table 3, Supplementary Table 3), all of which were also represented in male LVD patients (Figure 2, Table 3, Supplementary Table 2). However, only three of the 18 genes were unique to the female LVD cohort, including: LPAL2, ZFHX3, and CDKN2B which, although not previously reported as an LVD-associated risk gene, was also unique to LVD male patients, though the associated expressed exons and junctions differed between the sexes (Figure 4, Table 7A, Supplementary Tables 2, 3). Though not unique to LVD, one associated risk gene, HDAC9 (10, 26), was commonly represented in both sexes, albeit by differing exon and junction expression (Supplementary Tables 2, 3). One exon in ALDH2, PSR12011484, was commonly expressed in both sexes but was downregulated in male patients and upregulated in female patients (Table 6).

#### Differential Exon/Junction Usage in Small Vessel Disease/Lacunar Cause of Ischemic Stroke

Male SVD patients had 148 differentially expressed exons/junctions from 34 genes (Figure 2, Table 3, Supplementary Table 2). Similarly, females had 150 differentially expressed exons/junctions from 34 genes (Figure 2, Table 3, Supplementary Table 3). In male patients, four of the 34 SVD-associated genes were unique to that specific stroke cause: CYP4F2, GP1BA, solute carrier family four, member one (SLC4A1), and F13A1, which was also uniquely expressed in the male cohort (Figure 4A). In female patients, 12 of the 34 SVD-associated genes were unique to that stroke cause, including FGA, SLC22A3, both unique to the female cohort, and SLC4A1 which was also expressed in males, though by different exons and junctions (Figure 4, Tables 6, 7A). Twenty SVD genes were common between the sexes (Figure 2, Table 3, Supplementary Tables 2, 3). However, only eight were represented by 21 common exons/probesets, 18 of which had inverse expression levels between the sexes (Table 6).

Remarkably, six genes represented by differential exon and junction expression in male LVD patients were also represented in female SVD patients, including: F2, a LVD-risk associated gene, nitric oxide synthase (NOS1), paired like homeodomain 2 (PITX2), phospholipid phosphatase 3 (PLPP3), retinoic acid induced 1 (RAI1), and zinc finger C3HC-type containing 1 (ZC3HC1) (Figure 4, Table 7B). However, only one gene probeset, a junction, JUC17001562, in RAI1, was commonly expressed, albeit inversely, in both sexes (Table 7B).

### Time-Dependent Correlations in Expression of Stroke/VRF Risk Genes in Ischemic Stroke Patients

**Figure 5:**
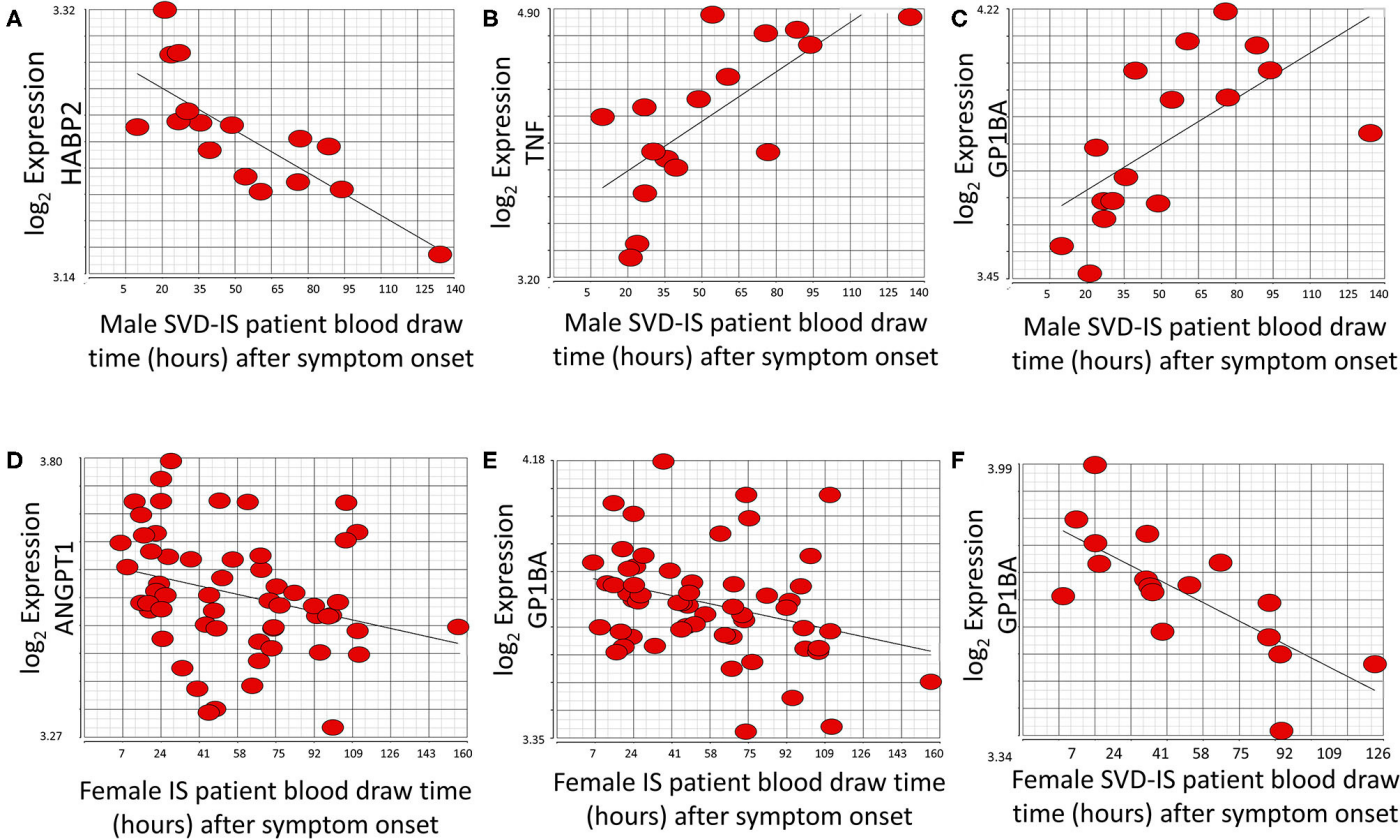
Genes that significantly correlate (*p* < 0.05; r > |0.4|) with time after symptom onset in male and female stroke patients. HABP2 **(A)** decreases, whereas both TNF **(B)**, and GP1BA **(C)** increase over time after symptom onset in male small vessel disease (SVD) ischemic stroke (IS) patients. ANGPT1 **(D)** and GP1BA **(E)** significantly decrease over time after symptom onset in all female IS patients. GP1BA expression in male patients increases over time **(C)** but decreases over time in female patients **(F)**. Actual statistical significance (*p* < 0.05) and correlation (r > |0.4|) values are presented in Supplementary Table 4. See methods section for statistical analysis protocol.

At the exon/junction level, 39 genes represented by 96 exons/junctions significantly correlated with time after all strokes in males (Supplementary Table 4). Hyaluronan binding protein 2 (HABP2; Figure 5A), tumor necrosis factor alpha (TNF) (Figure 5B), and GP1BA (Figure 5C) were correlated with time in male SVD patients. GP1BA and angiopoietin-1 (ANGPT1) negatively correlated with time after stroke in females at the gene level (Figures 5D,E). At the exon/junction level, 45 genes represented by 103 exons/junctions were significantly correlated with time after stroke in females (Supplementary Table 4). GP1BA was positively correlated with time in male SVD patients (Figure 5C) and negatively correlated with time in female SVD patients (Figure 5F). A probeset within an exon (PSR19008811) in APOE and a probeset spanning an exon-exon junction (JUC19001725) in LDLR had expression that was negatively correlated with time after stroke in male IS (Supplementary Table 4) but expression that was positively correlated with time in female IS patients (Supplementary Table 4).

## Discussion

Building on our previous results that alternatively spliced genes differ between stroke and vascular risk factor controls (13), we investigated differential expression of putative risk genes after ischemic stroke in male and female patients compared to vascular risk factor matched male and female control subjects. Thus, this is the first systematic study to demonstrate sexual dimorphism and differential alternative splicing (DAS) in expression of many GWAS-identified stroke risk genes in the blood of patients following the three main causes of ischemic stroke compared to control subjects. Notably, of the 71 genes studied, 62 showed differential exon/junction expression in large vessel disease (LVD), 48 showed differential exon/junction expression in small vessel disease (SVD) and 32 showed differential exon/junction expression in cardioembolism (CE). This demonstrates transcriptional responses to stroke in most loci that are dependent upon different causes of stroke.

The American Heart Association (AHA) it began identifying genes as risk factors in 2012 (50), adding more each year, though it still does not officially recognize all of the stroke subtype specific genes in this study (51). Given that this field of study continues to be very dynamic, in addition to the AHA reported risk genes (52), we included GWAS identified loci for ischemic stroke in general, as well as additional subtype risk genes and vascular risk factor genes (Table 1). In support of the AHA acknowledged genes, we found evidence for AS and/or differential expression of four of those genes. Specifically, in male patients ZFHX3, implicated in CE stroke (24, 49), and HDAC9, implicated in LVD stroke (24, 49), were significant for DAS. In female patients, ALDH2 implicated in SVD stroke (24), was significant for DAS in male LVD and female SVD, and SH2B adaptor protein three (SH2B3) also implicated in all/SVD stroke (1), had significant differential exons/junction expression in female SVD.

Though several other genes have been genetically associated with specific causes of stroke, their transcriptional post-stroke response was not necessarily specific to the risk-associated cause. Examples include SUPT3H/CDC5L which is implicated in LVD (25) yet showed differential exon/junction usage in male LVD and male SVD patients compared to control subjects. CDKN2A, implicated in LVD and all IS (25), was regulated in LVD in male and female patients and in male CE patients compared to control subjects. PITX2 which has been implicated in CE stroke (25), was differentially expressed in LVD in males and females and in female SVD. The CE associated gene, ZFHX3 (24, 49), was represented by differentially expressed exons/junctions in female LVD patients and male SVD patients. ABO, a putative LVD and CE risk gene (25), had differentially expressed exons/junctions in male and female patients with LVD and male CE etiologies. MMP12 implicated in LVD (25) was regulated in male and female LVD. NINJ2, implicated in all IS (25) was regulated in male and female LVD and SVD. NAA25, implicated in all IS (25), showed differential exon expression in female CE, female LVD, and female SVD and DAS in female CE. HDAC9, implicated in LVD and all IS (25), was significant for differential splicing not only in male LVD patients but also in male SVD patients, and showed differential exon expression in male and female LVD and male SVD.

We previously reported that several differentially expressed lncRNAs were in proximity to stroke risk and VRF genes (37). ADD1, a gene associated with increased stroke risk (27), was reported to be in the vicinity of a lncRNA with increased expression in female IS patients compared to controls (37) and was found in this study to be associated with DAS in male and female CE patients and male LVD patients. Additionally, LPA, significant for DAS in male CE and LVD patients and LPAL2—for DAS in male SVD patients, were in proximity to a lncRNA with increased expression in male IS patients compared to VRFCs (37). LPA also had differentially expressed exons in female CE, male LVD, and male and female SVD; and LPAL2 had differentially expressed exons/junctions in male and female LVD and male SVD. These results further support a need for better understanding of lncRNA functions in stroke biology.

WNK1 was of note since it had significant differential gene level expression in SVD, for DAS in male CE and SVD patients, and differential exon/junction expression in male CE, female LVD and both male and female SVD. This gene has previously been implicated in atherothrombotic stroke risk (42) but could not be validated in a follow-up study (35). Though we previously reported there were no changes in WNK1 expression, those studies were done at the gene level only, did not include separate sex analyses, and did not include small vessel disease/lacunar stroke (35). Thus, by assessing expression at different levels in both males and females with all three causes of stroke, a more complete picture of WNK1 expression was obtained in this study.

### Study Strengths and Limitations

The strengths of this study include investigating post-stroke differential sex- and cause-specific expression of risk genes at gene-, exon- and alternative splicing levels. However, there are several limitations of this study, including the non-exhaustive nature of our gene list due to the dynamic nature of this field of study. This is evidenced by a recently published GWAS paper identifying 22 novel risk associations (53) which should be considered in future research. However, in gene- and transcript-level analyses of 29 of the newly identified risk genes reported by Malik (53) and the AHA (54), we found sexually dimorphic differential expression for many of the genes when we compared stroke patients and control subjects (Table 8). Two genes, semaphorin 4A (SEMA4A) and pre-mRNA processing factor 8 (PRPF8), had significantly decreased expression (FC > |1.2|; FDR < 0.3) in male IS patients compared to VRFC, whereas there were no genes in the female comparison with significant differential expression. Five probesets representing five genes (castor zinc finger 1 (CASZ1), guanylate cyclase 1 soluble subunit alpha 3 (GUCY1A3), leucine rich repeats and calponin homology domain containing 1 (LRCH1), PRPF8, SH3, and PX domains 2A (SH3PXD2A) were significantly differentially expressed (FC > |1.2|; FDR < 0.3) and IS and VRFC females. In male IS vs. VRFC, 44 probesets, representing fourteen genes (ankyrin 2 (ANK2), CASZ1, FES proto-oncogene, tyrosine kinase (FES), GUCY1A3, interleukin enhancer binding factor 3 (ILF3), long intergenic non-protein coding RNA 1492 (LINC01492), polyamine modulated factor 1 (PMF1), PRPF8, SEMA4A, SH3PXD2A, solute carrier family 25 member 44 (SLC25A44), solute carrier family 44 member 2 (SLC44A2), zinc finger protein 318 (ZNF318) were significantly differentially expressed (FC > |1.2|; FDR < 0.3). LRCH1, a gene linked to cardiac mechanism, was represented only in women. In men, eight (ANK2, FES, ILF3, LINC01492, PMF1, SEMA4A, SLC25A44, SLC44A2, ZNF318) of the 14 represented genes were male-specific. Interestingly, the majority of differentially expressed probesets were exon-exon junctions in both men (56.5%) and in women (71.4%). These results, when combined with the differential expression in men of PRPF8, a gene that plays a distinct role in spliceosome assembly (55), suggest that AS may be playing a role in the differential expression of these newly identified stroke risk genes. Although we did not evaluate differential expression between the stroke causes, we found differential expression of genes that are associated with specific etiologies. LINC01492 is an identified risk gene for LVD (53, 54), PMF1, SLC25A44, and SEMA4A, a gene only recently linked with IS, are all associated with SVD (53, 54). These data reinforce the results of our more in-depth analyses that sex and etiology should both be considered in stroke studies.

**Table 8:** Significant differentially expressed genes, probesets and junctions of recently identified putative risk genes in male (M) and female (F) ischemic stroke (IS) patients compared to vascular risk factor control (VRFC) subjects.

**Gene symbol**	**Gene name**	**IS and/or** **stroke cause**	**DEG transcript Cluster** **or Exon (PSR)/** **Junction (JUC) probeset**	**FC (M-IS vs.** **M-VRFC)**	**FC (F-IS vs.** **F-VRFC)**
SEMA4A	Semaphorin 4A	IS	TC01001314	−1.21	
PRPF8	Pre-mRNA processing factor 8	IS	TC17000981	−1.31	
ANK2	Ankyrin 2	IS	JUC04004525	1.48	
			JUC04004528	1.25	
			JUC04004540	1.29	
			JUC04004550	1.28	
**CASZ1**	**Castor zinc finger 1**	**IS**	JUC01018095	−1.42	
			JUC01018101	1.36	
			JUC01018103		−1.23
			PSR01033752	1.22	
FES	FES proto-oncogene, tyrosine kinase	IS	JUC15004385	1.51	
			JUC15004401	−1.40	
			PSR15008257	−1.21	
			PSR15008282	−1.39	
			PSR15008295	−1.23	
**GUCY1A3**	**(GUCY1A1) guanylate cyclase 1 soluble subunit alpha 3**	**LVD**	JUC01018103		1.24
			PSR04010805	1.20	
**ILF3**	**Interleukin enhancer binding factor 3**	**IS**	JUC19001622	−1.46	
			PSR19002766	−1.22	
			**PSR19002774**	**−1.26**	**−1.23**
			PSR19002786	1.22	
LINC01492	Long intergenic non-protein coding RNA 1492	LVD	PSR09017801	1.29	
LRCH1	Leucine rich repeats and calponin homology domain containing 1	IS	PSR13002134		−1.21
PMF1	Polyamine modulated factor 1	ICH, SVD	JUC01010941	1.77	
			JUC01010953	2.16	
			PSR01020570	−1.32	
			PSR01020577	−1.27	
**PRPF8**	**pre-mRNA processing factor 8**	**IS**	JUC17007472	−1.55	
			JUC17007475	−1.65	
			JUC17007487	−1.33	
			JUC17007492	−1.49	
			JUC17007495		−1.47
			JUC17007499	−1.50	
			JUC17007502	−1.31	
			JUC17007505	−1.66	
			PSR17013135	−1.42	
SEMA4A	Semaphorin 4A	IS	JUC01010917	−1.56	
			PSR01020479	−1.25	
			PSR01020481	−1.42	
			PSR01020485	1.34	
			PSR01020505	−1.46	
			PSR01020507	−1.38	
			PSR01020508	−1.76	
			PSR01020509	−1.89	
**SH3PXD2A**	**SH3 and PX domains 2A**	**IS**	JUC10012342	1.20	
			JUC10012349		1.35
SLC25A44	solute carrier family 25 member 44	SVD	JUC01010929	−1.45	
		SVD	JUC01010935	−1.71	
SLC44A2	solute carrier family 44 member 2	IS	JUC19001593	−1.23	
		IS	PSR19002745	−1.21	
**ZCCHC14**	**zinc finger CCHC-type containing 14**	**IS**	**JUC16010494**	**−1.25**	**−1.22**
ZNF318	zinc finger protein 318	IS	JUC06009922	−1.56	
		IS	JUC06009932	−1.57	

*Significance: fold change (FC) > |1.2|; false discovery rate p-value (FDR) < 0.3. List of new putative risk genes was generated from Malik et al. (53) and Benjamin et al. (54). Bold font indicates probesets and genes expressed commonly in male and female subjects*.

Because stroke is known to affect immune responses, variations in gene expression could reflect changes in blood cell types. Therefore, future research should investigate gene expression of individual blood cell types after stroke. Additional limitations of this study include the relatively small sample size of some of the subgroups. The results need to be replicated. Alternative splicing was predicted rather than directly measured as can now be done with some sequencing systems. Race should be addressed in future studies, though our previous studies indicate small effects of race on gene expression following stroke (56).

Because of the putative involvement of these genes in stroke pathophysiology, this study investigates differential expression in the putative risk genes between male and female patients with ischemic stroke of different etiologies and male and female vascular risk factor matched controls but it is not our intention to suggest that sexual dimorphism exists only in the expression of these putative risk genes. Indeed, we expect that sexual dimorphism may affect non-stroke risk genes in a stroke-etiology specific manner. Future studies need to address the broader sexual dimorphism in stroke of different etiologies.

## Conclusion

This study provides evidence for a role of previous GWAS-identified genes beyond an increased risk for stroke and future research should investigate their role in post-stroke injury and/or recovery. Many of these genes are alternatively spliced between the three main causes of stroke and are sexually dimorphic. Strikingly, although few of the 71 genes studied pass GWAS statistical criteria (51), 70 of the 71 genes associated with stroke or stroke risk factors had differential expression in stroke patients compared to control subjects. Since sex and cause of stroke had such a large impact on these gene expression results, we suggest a need for stroke treatment trials and stroke genetic studies to be powered for inclusion of these variables as we search for more targeted stroke treatment and as society continues its trend toward more personalized medicine for patients.

## Data Availability Statement

The raw data supporting the conclusions of this article will be made available by the authors, without undue reservation.

## Ethics Statement

The studies involving human participants were reviewed and approved by University of California (UC), Davis Institutional Review Board, UC San Francisco Institutional Review Board and University of Alberta Health Research Ethics Board (Biomedical Panel). The patients/participants provided their written informed consent to participate in this study.

## Author Contributions

CD-A analyzed and interpreted the patients' array data. CD-A, FS, BS, and BA were major contributors in writing the manuscript. HH prepared samples and arrays. All authors read and approved the final manuscript.

## Conflict of Interest

The authors declare that the research was conducted in the absence of any commercial or financial relationships that could be construed as a potential conflict of interest.
