# Supplementary material for: Alternative Splicing of Putative Stroke/Vascular Risk Factor Genes Expressed in Blood Following Ischemic Stroke Is Sexually Dimorphic and Cause-Specific
Source: Front Neurol. 2020 Oct 22;11:584695. doi: 10.3389/fneur.2020.584695 (PMC7642687; doi:10.3389/fneur.2020.584695)
Supplement: Supplementary file 1 [file Data_Sheet_1.PDF]

Alternative Splicing of Putative Stroke/Vascular Risk Factor Genes Expressed in Blood Following Ischemic Stroke is Sexually Dimorphic and Cause-Specific

Cheryl Dykstra-Aiello PhD, Frank R Sharp MD, Glen C Jickling MD, Heather Hull BS, Farah Hamade BS, Natasha Shroff PhD, Marc Durocher BS, Xiyuan Cheng BS, Xinhua Zhan MD/PhD, DaZhi Liu PhD, Bradley P Ander PhD^ and Boryana S Stamova PhD^*

Supplementary Tables 1 - 4

Supplementary Table 1. Significant differentially expressed probesets between ischemic stroke (IS) patients and vascular risk factor matched controls (VRFC) in both sexes. Significance: $p<0.05$; fold change>|1.2|.

Gene Symbol	Affymetrix Exon (PSR)/ Junction (JUC) Probesets	MALE		FEMALE	
		p-value	Fold Change	p-value	Fold Change
ACE	PSR17009957	1.72E-02	1.30		
	JUC17005463			4.93E-02	-1.30
ADD1	JUC04000392				
	JUC04000392	4.08E-02	-1.34		
	JUC04000397	5.45E-03	-1.64		
	JUC04000415	2.47E-02	-1.28		
	PSR04001030	3.44E-02	-1.33		
ALDH2	JUC12019423	2.55E-02	-1.38		
	JUC12019428	1.63E-02	-1.35		
	JUC12019433	1.56E-02	-1.29		
	JUC12019445	2.54E-02	-1.36		
	JUC12019453	1.14E-02	-1.43		
	JUC12019459	3.08E-02	-1.60		
	JUC12019465	2.84E-03	-1.69		
	JUC12019474	4.07E-02	-1.47		
	PSR12011404	4.87E-04	-1.22		
	PSR12011407	1.50E-02	-1.34		
	PSR12011414	2.68E-03	-1.51		
	PSR12011458	4.90E-02	-1.22		
	PSR12011483	1.42E-02	-1.27		
	PSR12011486	2.71E-02	-1.30		
	JUC12019423	2.55E-02	-1.38		
ALOX5AP	JUC13000557	1.50E-02	-1.76		
	JUC13000558	7.39E-03	-1.70		
	JUC13000562	1.88E-02	-1.44		
	PSR13000960	6.96E-03	-1.53		
	PSR13000962	2.81E-03	-1.52		

	PSR13000964	8.21E-03	-1.52		
	PSR13000965	1.05E-02	-1.43		
	PSR13000966	4.36E-02	-1.35		
CDC5L	JUC06003334			3.62E-02	1.25
EPHX2	JUC08001494	4.5E-02	1.20		
F13A1	JUC06007367	1.65E-02	-1.55		
	JUC06007368	2.95E-02	-1.46		
	JUC06007369	2.64E-02	-1.48		
	JUC06007373	3.01E-03	-1.38		
	JUC06007376	2.71E-02	-1.74		
	JUC06007378	8.39E-03	-1.70		
	JUC06007379	2.77E-02	-1.36		
	JUC06007380	2.73E-03	-1.50		
	JUC06007381	4.14E-02	-1.39		
	JUC06007383	1.55E-02	-1.37		
	PSR06014791	8.72E-03	-1.54		
	PSR06014792	9.30E-03	-1.56		
	PSR06014793	1.96E-02	-1.47		
	PSR06014795	1.96E-02	-1.43		
	PSR06014796	1.28E-02	-1.52		
	PSR06014797	9.64E-03	-1.47		
	PSR06014798	4.15E-02	-1.36		
	PSR06014799	2.04E-02	-1.38		
	PSR06014800	1.15E-02	-1.41		
	PSR06014802	1.89E-02	-1.43		
	PSR06014803	3.14E-02	-1.40		
	PSR06014804	1.10E-02	-1.54		
	PSR06014805	6.65E-03	-1.43		
	PSR06014806	7.11E-03	-1.46		
	PSR06014808	9.98E-03	-1.41		
	PSR06014812	3.84E-02	-1.43		
F7	JUC13002800	4.93E-02	1.22		
	JUC13002805	1.33E-02	1.38		
GP1BA	PSR17000617	1.08E-02	-1.40		
	PSR17000619	1.04E-02	-1.61		
HDAC9	JUC07000798	3.99E-02	1.43		
	JUC07000815	1.83E-02	1.33		
	JUC07000816	4.98E-02	-1.46		

	JUC07000849	6.06E-03	-1.58		
	PSR07001801			4.84E-02	-1.25
	PSR07001824	3.40E-02	-1.37		
	PSR07001825	4.42E-02	-1.32		
IMPA2	JUC18000382	5.83E-03	-1.98		
	JUC18000384	1.61E-02	-1.73		
	JUC18000385	3.80E-03	-1.78		
	JUC18000390	1.42E-02	-1.65		
	PSR18000631	1.26E-02	-1.64		
	PSR18000632	4.52E-03	-1.38		
	PSR18000635	9.69E-03	-1.64		
	PSR18000636	1.44E-02	-1.68		
	PSR18000637	2.65E-03	-1.73		
ITGB3	JUC17018721	2.64E-02	1.65		
	JUC17018745			8.05E-03	1.35
	PSR17007462			3.97E-02	1.31
	PSR17007464			3.28E-02	1.25
	PSR17007469			3.89E-02	1.25
LDLR	JUC19001726	3.17E-02	-1.39		
LPL	JUC08000810	4.17E-03	1.42		
LTA	JUC06001489	8.62E-03	1.43		
LTC4S	JUC05019516	2.69E-02	-1.24		
	JUC05019519	2.01E-02	-1.63		
	PSR05015363	1.53E-02	-1.35		
	PSR05015365	9.45E-03	-1.57		
MTHFR	JUC01018273	2.09E-03	-1.62		
	JUC01018286	*8.20E-04*	*1.51*	4.62E-02	1.23
NINJ2	JUC12007875	1.60E-02	-1.84		
	JUC12007877	3.26E-02	-1.33		
	PSR12014329	1.38E-02	-1.67		
	PSR12014331	2.82E-02	-1.62		
	PSR12014332	1.02E-02	-1.84		
	PSR12014335	1.06E-02	-1.65		
	PSR12014336	7.48E-03	-1.66		
NOS1	JUC12014780	2.74E-02	1.44		
PCSK9	JUC01005610	6.43E-03	-1.76		
PDE4D	JUC05010000	1.07E-02	-1.80		
	JUC05010011	2.11E-02	-1.65		

	PSR05019373	2.16E-02	-1.55		
	PSR05019374	4.80E-02	-1.50		
	PSR05019376	1.50E-02	-1.39		
	PSR05019383	4.68E-02	-1.32		
PEMT	JUC17009234	1.94E-02	1.30		
	JUC17009237	1.56E-02	-1.74		
	JUC17009240	8.13E-04	1.21		
	JUC17009249	2.20E-02	-1.34		
	JUC17009251	3.84E-02	-1.29		
	PSR17016157	3.75E-02	-1.43		
	PSR17016172	2.11E-02	-1.33		
PRKCH	JUC14002038			3.68E-02	-1.29
	JUC14002043			3.92E-02	-1.46
	JUC14002046			4.90E-02	-1.46
	PSR14004098			4.66E-02	-1.37
	PSR14004100			3.85E-02	-1.33
SGK1	JUC06013228	3.66E-02	1.23		
	JUC06013231	4.17E-02	-1.46		
	PSR06026689	2.68E-02	-1.33		
	PSR06026704	3.39E-02	-1.36		
SH2B3	JUC12006316	1.49E-03	1.40		
	JUC12006317	4.85E-02	1.25		
	JUC12006319	6.94E-03	-1.70		
	PSR12011385	4.96E-02	-1.28		
	PSR12011386	1.19E-02	-1.29		
	PSR12011387	4.76E-02	-1.32		
SMARCA4	JUC19001672	4.79E-03	-1.36		
	JUC19001685	1.02E-02	1.39		
	JUC19001699	4.17E-03	-1.49		
	JUC19001708	1.59E-02	-1.26		
	JUC19001709			4.89E-02	-1.31
	PSR19002909	3.38E-02	-1.26		
SORT1	JUC01024739			1.91E-02	2.45
	PSR01045962	4.74E-02	-1.27		
SUPT3H	PSR06021220	2.32E-02	-1.31		
WNK1	JUC12000095	2.67E-03	-1.78		
	JUC12000105	4.62E-02	-1.60		
	JUC12000121	2.05E-02	-1.68		

	JUC12000131	4.88E-02	-1.33	
	JUC12000132	2.19E-02	-1.65	
	PSR12000128	3.25E-02	-1.36	
	PSR12000131	2.16E-02	-1.34	
	PSR12000163	2.79E-02	-1.53	
	PSR12000164	4.29E-02	-1.60	
	PSR12000165	2.33E-02	-1.36	
	PSR12000167	4.05E-02	-1.48	
	PSR12000173	5.51E-03	-1.33	
	PSR12000175	4.63E-02	-1.31	
	PSR12000178	2.65E-02	-1.34	
WNK1-NC	PSR12028413	3.26E-02	-1.23	
ZC3HC1	JUC07014252	2.43E-02	1.32	
ZFHX3	JUC16009974	5.13E-03	-1.99	
	JUC16009977	2.73E-02	-1.30	
ZPR1	JUC11014794	2.95E-02	1.25	
	JUC11014803	4.06E-02	1.23	

Supplementary Table 2. Significant differentially expressed probesets within the three main causes of ischemic stroke versus vascular risk factor matched controls in the <u>male cohort</u>. Bold font indicates the 21 sex-specific representative genes. Significance: $p<0.05$; fold change (FC) > |1.2|. CE- cardioembolism; LVD-large vessel disease IS; SVD-small vessel disease/lacunar.

Gene Symbol	Exon (PSR) and Junction (JUC) Probesets	CE		LVD		SVD	
		p-value	FC	p-value	FC	p-value	FC
**ABO**	JUC09011574	4.25E-02	-1.23				
	JUC09011575	3.61E-02	-1.22				
	PSR09021554			1.23E-02	1.58		
	PSR09021565			3.45E-03	2.06		
ACE	JUC17005449			4.21E-02	1.49		
	JUC17005450					1.25E-02	-1.37
	JUC17005463			2.09E-02	-3.10		
	JUC17005474					3.32E-02	-2.54
	JUC17005489			1.44E-02	1.70		
	PSR17009932			2.49E-02	1.60		
	PSR17009946			2.68E-03	3.31		
	PSR17009957			6.45E-03	2.62		
ADD1	JUC04000389			4.27E-02	-3.34		
	JUC04000392					1.94E-02	-1.69
	JUC04000397			1.78E-03	-5.73	1.02E-02	-2.04
	JUC04000400			3.02E-02	-1.84		
	JUC04000401			3.00E-02	-1.84		

	JUC04000402			3.35E-03	-2.27	3.72E-02	-1.34
	JUC04000406			4.36E-02	2.56		
	JUC04000410			2.37E-02	-1.79		
	JUC04000412			1.66E-02	-4.31		
	JUC04000413	1.25E-02	2.08	2.59E-02	-3.22	2.03E-02	-1.82
	PSR04000982			2.76E-02	1.64		
	PSR04000988			1.23E-02	-2.33		
	PSR04001000			3.91E-02	-1.58		
	PSR04001002			1.72E-03	-2.45		
	PSR04001005			1.47E-03	-2.67		
	PSR04001007			5.40E-03	-2.00		
	PSR04001008	3.51E-02	-1.23				
	PSR04001010			3.37E-02	-1.62		
	PSR04001011			4.38E-02	1.64		
	PSR04001014			1.06E-02	-2.10		
	PSR04001017					2.75E-02	-1.40
	PSR04001018			3.78E-03	-3.81		
	PSR04001020			2.10E-02	-1.70		
	PSR04001025					3.11E-02	-1.42
	PSR04001026					1.47E-02	-1.55
	PSR04001029			2.46E-02	-1.41		
	PSR04001030			1.38E-02	-3.00	3.76E-02	-1.56
	PSR04001032					3.22E-02	-1.69
	PSR04001037			2.87E-02	-2.58	3.02E-02	-1.59
	PSR04001038			3.65E-02	-1.83	9.87E-03	-1.46
AIM1	JUC06004671					1.53E-02	1.44
	JUC06004685					1.73E-02	1.60
	JUC06004686					4.52E-02	1.28
	JUC06004691			4.80E-03	-2.80		
	PSR06009989					2.09E-02	1.30
	PSR06009994			1.47E-02	-2.15		
	PSR06009995			3.06E-02	-1.58		
	PSR06009997			3.38E-02	-1.91		
	PSR06010000			1.38E-02	-2.08		
	PSR06010008			9.91E-03	-1.94		
ALDH2	JUC12019433	4.38E-02	-1.47				
	JUC12019434	2.39E-02	-1.21				
	JUC12019439			5.79E-03	3.87		
	JUC12019449					3.49E-02	-1.54
	JUC12019451			3.86E-02	-1.63		
	JUC12019461					4.66E-02	-1.82
	JUC12019465					7.89E-03	-2.30
	JUC12019470			1.22E-02	-2.19		
	JUC12019472					4.02E-02	-1.94
	JUC12019473			2.06E-02	3.02		
	PSR12011398	3.21E-02	-1.43				
	PSR12011406			3.16E-02	-2.21		
	PSR12011407			1.23E-02	-2.56		
	PSR12011408			4.66E-02	-1.66		
	PSR12011414			1.46E-03	-4.16		
	PSR12011419			3.24E-02	-2.08		

	PSR12011429			7.27E-03	-1.94		
	PSR12011431			4.56E-02	-1.45		
	PSR12011437			2.61E-02	-1.51		
	PSR12011464			2.73E-02	1.67		
	PSR12011471			2.55E-02	-3.89		
	PSR12011472			3.21E-02	-1.48		
	PSR12011484			3.39E-02	-1.60		
	PSR12011488					3.11E-02	-1.81
**ALOX5AP**	JUC13000558					4.12E-02	-1.93
	JUC13000559			2.64E-02	2.54		
	PSR13000960					4.79E-02	-1.66
	PSR13000962					2.29E-02	-1.67
ANGPT1	JUC08010138			1.13E-02	1.79		
	JUC08010141			3.39E-02	-1.45		
**APOC1**	PSR19008817			1.93E-02	1.47		
**APOE**	JUC19005251			8.24E-04	4.63		
APOL2	JUC22005130			6.75E-04	-14.83		
	JUC22005136			4.17E-03	4.32		
	JUC22005140			2.31E-03	-3.29		
	JUC22005141					2.53E-02	1.42
	JUC22005142			3.84E-02	1.68		
	PSR22012365	3.35E-02	-1.61	4.77E-02	-1.98		
	PSR22012366			4.20E-02	-1.98		
	PSR22012368			2.01E-03	-4.08		
	PSR22012369	1.65E-02	-1.48				
	PSR22012378					2.07E-02	1.58
	PSR22012379	2.23E-02	-1.98			1.38E-02	2.00
	PSR22012380	1.95E-02	-1.84				
	PSR22012381			3.03E-02	-2.55		
	PSR22012383			4.34E-02	2.18		
**CDC5L**	JUC06003329		4.25E-02	-2.61			
	PSR06007539				4.45E-02	1.37	
**CDKN2A**	JUC09006948	4.52E-02	-1.53				
	JUC09006951			2.74E-02	1.73		
	PSR09012771			3.45E-02	1.51		
CDKN2B	JUC09006956		2.97E-02	3.18			
**CRP**	JUC01027710		2.26E-02	1.74			
	PSR01052887		4.04E-02	1.63			
**CYP11B2**	JUC08011562		4.00E-03	1.75			
CYP4A11	JUC01021969	4.26E-02	1.34				
	JUC01021971			4.71E-02	1.98		
	JUC01021975			2.71E-02	-1.43		
	JUC01021984			1.73E-03	2.14		
	JUC01021987			3.24E-02	2.17		
	JUC01021989			1.11E-03	6.19		
	PSR01041072			2.48E-06	2.36		
	PSR01041094	3.60E-02	-1.44	4.38E-02	1.92		
CYP4F2	JUC19010335					3.09E-02	-1.28
DDAH1	JUC01023617			2.01E-02	-1.42		
	PSR01044026			3.57E-04	2.35		

	PSR01044034			1.88E-02	1.55		
	PSR01044036			1.29E-02	1.82	1.59E-02	1.33
	PSR01044044			2.95E-03	1.87		
EPHX2	JUC08001470			1.29E-03	2.59		
	JUC08001478			3.89E-02	2.76		
	JUC08001485	1.58E-02	1.46				
	JUC08001488			1.18E-03	2.60		
**F13A1**	JUC06007367					6.54E-04	-3.23
	JUC06007368					2.66E-03	-2.70
	JUC06007369					2.42E-03	-2.71
	JUC06007370					4.63E-02	-1.32
	JUC06007371					6.77E-04	-3.84
	JUC06007372					3.45E-02	-2.06
	JUC06007373					2.02E-04	-2.06
	JUC06007375					1.34E-02	-2.17
	JUC06007376					1.19E-03	-4.36
	JUC06007378					3.36E-03	-2.91
	JUC06007379					7.17E-03	-2.04
	JUC06007380					3.39E-04	-2.40
	JUC06007381					7.57E-03	-2.34
	JUC06007383					1.40E-03	-2.18
	PSR06014790					2.09E-02	-2.33
	PSR06014791					9.71E-04	-2.71
	PSR06014792					4.72E-03	-2.43
	PSR06014793					7.77E-04	-2.76
	PSR06014795					1.10E-03	-2.55
	PSR06014796					1.65E-03	-2.75
	PSR06014797					1.88E-04	-2.82
	PSR06014798					3.57E-03	-2.35
	PSR06014799					2.15E-03	-2.26
	PSR06014800					1.15E-03	-2.30
	PSR06014802					1.04E-03	-2.61
	PSR06014803					3.68E-03	-2.39
	PSR06014804					8.57E-04	-2.97
	PSR06014805					1.72E-03	-2.15
	PSR06014806					3.55E-04	-2.48
	PSR06014808					1.17E-03	-2.22
	PSR06014810					2.25E-02	-2.20
	PSR06014812					1.61E-03	-2.76
	PSR06014814					2.70E-03	-2.47
**F13B**	JUC01029311					2.73E-02	-2.50
	JUC01029312			1.28E-03	11.73		
	PSR01056139			1.24E-02	1.50		
F2	PSR11005085			1.37E-02	2.35		
**F5**	JUC01028341			1.49E-02	3.81		
	JUC01028352	2.71E-02	2.42				
	JUC01028356	1.11E-02	2.89				
	PSR01054353	3.86E-02	1.41				
	PSR01054361	3.00E-02	2.35				
	PSR01054379	2.83E-02	1.94				

	PSR01054382	4.29E-02	1.79				
	PSR01054383	2.46E-02	1.65				
	PSR01054385			2.01E-02	1.93		
F7	JUC13002795			1.94E-02	3.68		
	JUC13002796			3.33E-02	1.90		
	JUC13002797	3.15E-02	1.29	2.97E-04	2.02		
	JUC13002800			4.62E-02	1.95		
	PSR13004632			4.74E-02	2.25		
	PSR13004634			2.04E-02	1.95		
	PSR13004644			2.86E-02	1.90		
	PSR13004647			6.44E-03	4.65		
FGB	JUC04005756			1.02E-02	1.63		
	JUC04005761	2.77E-02	-1.22				
	JUC04005765			1.22E-02	4.23		
	PSR04010713			1.34E-02	2.34		
	PSR04010731			1.24E-02	-1.50		
GP1BA	PSR17000619					3.51E-02	-1.99
**HABP2**	JUC10005528			4.36E-02	1.35		
	JUC10005531			3.36E-02	1.39		
HDAC9	JUC07000793					4.19E-02	-1.22
	JUC07000798			2.50E-02	3.36		
	JUC07000803			1.83E-02	1.60		
	JUC07000807			1.90E-02	2.00		
	JUC07000810	2.73E-02	-1.50				
	JUC07000825			2.91E-02	1.65		
	JUC07000842			1.63E-02	-2.57		
	JUC07000844			3.28E-02	2.64		
	JUC07000847			2.30E-02	1.65		
	PSR07001773					2.57E-02	1.32
	PSR07001809			3.03E-02	-3.18		
	PSR07001848			2.97E-05	2.91		
**IL1A**	JUC02018641		2.44E-03	-3.61			
IMPA2	JUC18000382			7.25E-03	-6.57	2.53E-03	-3.13
	JUC18000384					5.09E-03	-2.83
	JUC18000385			1.26E-02	-4.25	3.67E-03	-2.39
	JUC18000386	3.47E-02	1.21	8.44E-05	2.49		
	JUC18000387	3.93E-02	2.02			4.34E-02	-1.81
	JUC18000388					8.33E-04	-3.41
	JUC18000389			6.51E-03	2.14		
	JUC18000390					1.19E-03	-2.68
	JUC18000391					4.48E-02	-1.71
	PSR18000628					2.86E-02	-1.78
	PSR18000630					2.68E-02	-1.80
	PSR18000631					1.92E-03	-2.62
	PSR18000632			8.58E-03	-2.37	2.78E-03	-1.66
	PSR18000633					3.64E-02	-1.39
	PSR18000635			1.66E-02	-3.68	1.03E-03	-2.74
	PSR18000636			3.31E-02	-3.91	1.25E-02	-2.25
	PSR18000637					1.85E-03	-2.45
ITGA2B	JUC17011929			4.17E-02	2.18		

	JUC17011950				4.22E-02	-2.00
	JUC17011952				3.74E-02	-3.30
	JUC17011954		2.84E-04	1.96		
	PSR17021289		2.60E-02	2.52		
	PSR17021306				3.68E-02	-2.58
	JUC17018701		3.45E-02	3.11		
	JUC17018705				2.95E-02	-2.51
	JUC17018706				1.98E-02	-2.38
	JUC17018710				2.56E-02	-2.76
	JUC17018711				1.95E-02	-3.31
	JUC17018714				3.88E-02	-2.98
	JUC17018721				2.05E-02	2.52
	JUC17018724		2.29E-02	3.14		
ITGB3	JUC17018731				2.30E-02	1.49
	JUC17018736		2.06E-02	3.39		
	JUC17018738		2.47E-02	1.98		
	JUC17018745				1.87E-02	1.66
	PSR17007419				3.07E-02	-2.56
	PSR17007420				4.78E-02	-2.70
	PSR17007442				4.86E-02	-1.89
	PSR17007480				2.01E-02	1.30
	PSR17007481				1.59E-02	1.38
	PSR17007482				2.67E-03	1.40
	JUC19001720		2.18E-02	-1.51		
	JUC19001726		3.56E-02	-2.89		
	JUC19001729		3.20E-02	-1.57	2.90E-02	-1.27
	JUC19001739		2.83E-02	-1.58		
	JUC19001740		1.82E-02	-2.81		
LDLR	PSR19002951		3.75E-02	-1.74		
	PSR19002957		3.98E-02	-2.12		
	PSR19002958		2.16E-02	-2.64		
	PSR19002963		2.62E-02	-2.88		
	PSR19002965		1.96E-02	-2.11		
	PSR19002970		3.65E-03	2.06		
	JUC06014304		4.05E-04	1.21		
	JUC06014308		2.53E-02	1.67		
	JUC06014309		2.20E-03	3.22		
	JUC06014319		9.61E-03	2.40		
	JUC06014349		1.84E-02	1.66		
	JUC06014355		1.84E-02	1.66		
	JUC06014360		1.84E-02	1.66		
LPA	JUC06014364		1.84E-02	1.66		
	JUC06014366		1.84E-02	1.66		
	JUC06014367		1.25E-02	1.74		
	JUC06014374		1.25E-02	1.74		
	JUC06014402		7.70E-03	1.56		
	PSR06028639		5.62E-03	1.61		
	PSR06028649		1.04E-04	2.11		
	PSR06028653		1.04E-03	3.54		
	PSR06028655		4.76E-03	4.02	3.71E-02	1.60

	PSR06028657			4.76E-03	4.02	3.71E-02	1.60
	PSR06028659			4.76E-03	4.02	3.71E-02	1.60
	PSR06028661			1.04E-03	3.54		
	PSR06028663			4.76E-03	4.02	3.71E-02	1.60
	PSR06028665			4.76E-03	4.02	3.71E-02	1.60
	PSR06028667			4.39E-05	1.96		
LPAL2	JUC06014295			8.93E-03	4.07	2.90E-02	1.57
	PSR06028618			4.54E-02	1.92	3.33E-02	1.43
	PSR06028623					6.54E-03	1.35
LPL	JUC08000810			1.82E-02	2.81		
	PSR08001503			4.29E-02	1.81	1.89E-02	-1.31
	PSR08001517					5.24E-03	1.33
**LTA**	JUC06001485			1.56E-02	1.76		
**LTC4S**	JUC05019516			1.42E-02	-2.19		
	JUC05019522			2.00E-03	2.58	2.30E-02	1.36
	JUC05019525			2.79E-02	1.59		
	PSR05015363			1.02E-02	-2.79		
	PSR05015365			2.31E-02	-3.94		
	PSR05015366			6.29E-03	-2.09	1.90E-02	-1.42
	PSR05015368			3.43E-02	-2.52		
**MMP12**	JUC11014233			1.62E-02	2.67		
	JUC11014235			1.73E-02	1.43		
	JUC11014240			2.56E-04	1.87		
	JUC11014242			1.78E-02	3.55		
	JUC11014243			1.13E-05	2.84		
MTHFR	JUC01018273			2.78E-02	-3.48	2.16E-02	-1.84
	JUC01018281			4.85E-02	2.18		
	JUC01018284			1.36E-02	3.55		
	JUC01018286	1.72E-02	1.70				
	PSR01034075			3.63E-03	-2.69		
NAA25	JUC12014403			2.11E-02	-3.01		
	JUC12014404			4.32E-02	-2.22		
	JUC12014418			2.07E-03	3.00		
	JUC12014425			4.06E-02	-3.05		
	PSR12025872			1.73E-03	-3.35		
	PSR12025875			5.50E-04	-4.18		
	PSR12025876			9.95E-04	-2.39		
	PSR12025877			1.66E-02	-1.74		
	PSR12025880			3.59E-03	-2.07		
	PSR12025884			2.63E-02	-1.92		
	PSR12025888			7.43E-03	-2.03		
NINJ2	JUC12007875			2.89E-03	-11.95		
	PSR12014328			1.45E-02	-5.74		
	PSR12014332			1.15E-02	-6.98		
	PSR12014335			8.43E-03	-5.47		
	PSR12014336			2.44E-02	-4.18	4.98E-02	-1.92
	PSR12014337					1.46E-02	-1.44
NOS1	JUC12014770			8.87E-04	2.60		
	JUC12014780			5.92E-04	5.81		
	JUC12014783			2.97E-02	1.46		

	JUC12014789			2.45E-03	1.67		
	PSR12026409			3.90E-02	1.40		
	PSR12026415			5.83E-03	2.64		
NOS3	JUC07007462					1.16E-02	-1.42
	JUC07007464	4.52E-02	2.19				
	JUC07007466			2.34E-02	4.12		
	JUC07007467			2.75E-02	2.62		
	PSR07015425			1.09E-03	1.84		
	PSR07015433			1.40E-02	1.65		
	PSR07015446			1.87E-03	2.02		
**NPY**	JUC07001180			3.31E-02	2.04		
	JUC07001181			7.12E-04	2.24		
	PSR07002429			1.06E-03	1.68		
PCSK9	JUC01005608			1.48E-02	-2.13		
	JUC01005610			4.68E-03	-5.15	5.31E-04	-2.75
	JUC01005618			3.48E-02	2.21		
	PSR01010941			2.31E-04	2.21		
PDE4D	JUC05009956			4.42E-02	1.47		
	JUC05009960			3.73E-02	-2.98		
	JUC05009969			4.45E-02	3.09		
	JUC05009971			4.84E-02	2.26		
	JUC05009972			1.06E-02	1.48		
	JUC05009975			5.73E-03	2.83		
	JUC05009976			1.65E-02	1.85		
	JUC05009979			4.14E-02	-1.56		
	JUC05009990			4.69E-04	3.40		
	JUC05009995			2.79E-02	-2.06		
	JUC05010002			2.06E-02	-1.42		
	JUC05010010			8.94E-03	-1.62		
	PSR05019337	4.57E-02	1.40				
	PSR05019343			5.28E-03	2.23		
	PSR05019349			4.37E-02	-1.86		
	PSR05019360			9.61E-03	-1.75		
	PSR05019372			2.60E-02	3.22		
	PSR05019383	4.88E-02	-1.65				
	PSR05019390					1.81E-02	1.48
	PSR05019404			2.94E-03	3.49		
PDGFC	JUC04012326			5.60E-04	4.48		
	JUC04012328			2.74E-03	2.56		
	PSR04023300					3.98E-02	-1.56
PEMT	JUC17009234			4.34E-02	2.09		
	JUC17009235			4.76E-03	2.04		
	JUC17009237					2.04E-02	-2.40
	JUC17009246			2.72E-03	1.70		
	JUC17009251					1.29E-02	-1.64
	PSR17016157					4.81E-02	-1.78
	PSR17016158					4.63E-02	-1.25
	PSR17016167					2.92E-02	-1.54
	PSR17016173			7.48E-03	1.90		
	PSR17016175			7.63E-03	-1.65	3.91E-03	-1.35

PITX2	JUC04010982			1.68E-02	1.74		
PLPP3	PSR01042365			2.24E-02	2.10		
PON1	JUC07021478			2.56E-02	1.87		
	PSR07024550	3.49E-02	-1.36				
PRKCH	JUC14002035			4.85E-02	-2.07		
	JUC14002049			2.91E-02	-4.23		
	PSR14004093			3.66E-02	-2.77		
	PSR14004094			3.47E-02	-3.49		
	PSR14004099			3.02E-02	-2.59		
	PSR14004100			8.98E-03	-3.35		
	PSR14004102			1.59E-02	-3.69		
	PSR14004103			1.14E-02	-3.85		
	PSR14004105			5.73E-03	-3.50		
**PTGIS**	JUC20006659			3.78E-02	1.87		
RAI1	JUC17001559			1.60E-02	-2.30		
	JUC17001562			9.96E-03	3.86		
	PSR17002766			3.32E-02	1.55		
**SERPINE1**	PSR07010042			1.79E-02	1.67		
SGK1	JUC06013218			1.85E-02	1.99		
	JUC06013232			2.33E-02	3.14		
	JUC06013241			3.04E-03	1.86		
	PSR06026689					3.09E-02	-1.61
	PSR06026692					2.73E-02	-1.27
	PSR06026704					3.27E-02	-1.77
	PSR06026736			2.74E-02	1.83		
SH2B3	JUC12006316			3.64E-02	1.91		
	JUC12006317			1.45E-02	2.44		
SLC4A1	JUC17011883					4.53E-02	-1.53
SMARCA4	JUC19001668			6.96E-06	3.06		
	JUC19001671			8.26E-03	4.66		
	JUC19001673					7.27E-03	-1.68
	JUC19001676					3.98E-02	-1.39
	JUC19001683					1.96E-02	1.74
	JUC19001685			1.60E-02	2.74		
	JUC19001686			4.99E-02	-2.53		
	JUC19001689			2.01E-02	-3.86		
	JUC19001693			2.49E-02	-2.51		
	JUC19001698			4.51E-03	-3.80		
	JUC19001699			1.10E-02	-3.20	2.66E-02	-1.67
	JUC19001702			4.75E-02	-1.40		
	JUC19001703					2.49E-02	-2.19
	JUC19001708					5.57E-03	-1.68
	PSR19002889			1.25E-02	1.51		
	PSR19002891			2.17E-04	2.59		
	PSR19002909			4.97E-03	-2.73		
	PSR19002914			2.41E-02	-2.28		
	PSR19002923			4.07E-04	-4.10		
	PSR19002924			9.28E-04	-3.07		
	PSR19002925			1.26E-02	-1.67		
	PSR19002940			4.91E-03	-2.64		

	PSR19002941			2.05E-02	-4.66		
SORT1	JUC01024731			3.35E-02	-4.05		
	JUC01024735			6.87E-03	-5.27		
	PSR01045953			3.35E-02	-2.97		
	PSR01045958			2.41E-02	-2.67		
	PSR01045960			4.34E-02	-3.00		
	PSR01045961			1.92E-02	-2.22	4.17E-02	-1.43
	PSR01045962			3.62E-03	-3.18		
	PSR01045984					1.04E-02	-1.34
**SUPT3H**	PSR06021227			3.36E-02	1.45		
	PSR06021236			2.28E-02	1.81		
**TNF**	JUC06001492	5.33E-03	-1.61				
WNK1	JUC12000095					4.99E-03	-2.62
	JUC12000103			5.31E-03	7.36		
	JUC12000105			3.89E-02	-4.84		
	JUC12000110			4.27E-02	2.25		
	JUC12000116			9.86E-04	1.94		
	JUC12000121					2.41E-02	-2.43
	JUC12000128			2.49E-03	-4.16		
	JUC12000131					4.54E-02	-1.61
	JUC12000132			3.67E-02	-5.12		
	PSR12000128					3.31E-02	-1.71
	PSR12000131					2.29E-02	-1.70
	PSR12000154			4.20E-02	-2.81		
	PSR12000163					4.39E-02	-1.87
	PSR12000165					2.92E-02	-1.66
WNK1-lncRNA	PSR12028413					4.65E-02	-1.39
ZC3HC1	JUC07014252			4.89E-03	3.28		
	JUC07014254			3.18E-02	2.42		
	PSR07028796			5.44E-03	-1.76		
	PSR07028800			1.05E-02	1.61		
ZFHX3	JUC16009970			6.08E-03	2.62		
	JUC16009971			4.87E-03	2.19		
	JUC16009973			3.62E-02	1.94		
	JUC16009974					3.78E-02	-2.34
	JUC16009977					2.91E-02	-1.57
ZPR1	JUC11014792			2.38E-02	1.49		
	JUC11014798			2.69E-02	-2.17		
	JUC11014799	1.55E-02	1.74				
	JUC11014801			1.59E-02	-3.64		

Supplementary Table 3. Significant differentially expressed probesets within the three main causes of ischemic stroke versus vascular risk factor matched controls in the female cohort. Bold font indicates the 21 sex-specific representative genes. Significance: $p<0.05$; fold change (FC) > |1.2|. CE- cardioembolism; LVD-large vessel disease IS; SVD-small vessel disease/lacunar.

Gene Symbol	Exon (PSR) and Junction (JUC) Probesets	CE		LVD		SVD	
		**p-value**	**FC**	**p-value**	**FC**	**p-value**	**FC**

ACE	JUC17005445					2.75E-02	-1.45
	JUC17005450			9.59E-03	1.62		
	JUC17005463	1.34E-02	-1.94				
	JUC17005472	6.24E-03	1.61	1.03E-02	1.79		
ADD1	JUC04000389	2.38E-02	-2.18			4.51E-02	1.74
	JUC04000397	1.48E-02	-2.16				
	JUC04000400	1.52E-02	-1.39				
	JUC04000402	4.39E-02	-1.36			4.40E-02	1.30
	JUC04000407	9.15E-03	-1.36				
	JUC04000408					4.53E-02	1.71
	JUC04000410	1.63E-02	-1.35				
	JUC04000412	7.12E-03	-2.36				
	JUC04000414	3.79E-02	-1.32				
	JUC04000415					1.98E-03	1.59
	PSR04001002					2.88E-02	1.30
	PSR04001005	3.77E-02	-1.31			3.26E-02	1.27
	PSR04001016			3.33E-03	-1.64		
	PSR04001017	4.37E-02	-1.36				
	PSR04001018	8.68E-03	-1.63				
	PSR04001021	4.09E-03	-1.79			2.15E-02	1.44
	PSR04001022					3.07E-02	1.31
	PSR04001025					3.97E-02	1.26
	PSR04001026					1.03E-02	1.42
	PSR04001030					3.06E-02	1.45
	PSR04001032					2.40E-02	1.42
	PSR04001033					5.80E-03	1.75
	PSR04001035					4.59E-02	1.43
	PSR04001038					3.65E-02	1.27
AIM1	JUC06004671	1.93E-03	-1.71				
	JUC06004677	2.44E-02	1.35				
	JUC06004691	3.76E-02	-1.53				
	PSR06009991			7.76E-03	-2.03		
	PSR06010009	2.19E-02	-1.38				
ALDH2	JUC12019439					2.57E-02	-1.63
	JUC12019452					3.08E-02	1.33
	JUC12019458			5.60E-03	-1.39		
	JUC12019464					2.24E-02	-1.42
	JUC12019466					4.88E-03	-1.73
	JUC12019471	3.32E-02	1.71				
	PSR12011481	4.60E-02	1.62				
	PSR12011484			4.39E-02	1.47		
ANGPT1	PSR08019935	3.41E-02	1.26				
APOL2	JUC22005133	3.62E-02	-1.40				
	JUC22005136					3.59E-02	-1.52
	PSR22012365	2.90E-02	-1.57				
	PSR22012366	3.04E-02	-1.49				
	PSR22012370			4.25E-02	2.13		

	PSR22012374					1.74E-02	1.45
	PSR22012379	2.57E-02	-1.82				
	PSR22012386	3.26E-02	1.38				
	PSR22012387					3.96E-02	-1.29
CDKN2B	PSR09012783			4.80E-03	1.47		
CYP4A11	JUC01021974					2.22E-02	-1.33
CYP4F2	JUC19010335	2.19E-02	1.33				
	PSR19017731					3.10E-02	-1.32
DDAH1	JUC01023618					9.83E-03	-1.60
	PSR01044037	4.91E-02	1.30				
EPHX2	JUC08001468			3.16E-02	-1.28		
	JUC08001485					1.86E-02	1.40
	JUC08001488					3.48E-02	-1.31
	PSR08003009					1.04E-02	1.51
	PSR08003010					4.03E-03	1.70
	PSR08003013					2.23E-03	1.63
	PSR08003014					2.81E-02	1.80
F2	PSR11005066					1.24E-02	-1.35
F7	JUC13002792					3.82E-02	-1.46
	PSR13004647					3.17E-02	-1.68
	PSR13004653	2.00E-02	1.35				
**FGA**	JUC04012259				9.63E-03	-1.69	
FGB	JUC04005759	1.98E-02	1.27				
GP1BA	JUC17007936	3.15E-02	1.42			3.98E-02	-1.31
HDAC9	PSR07001774			1.69E-02	1.34		
	PSR07001797	3.70E-02	-1.25				
	PSR07001801	5.76E-03	-1.68				
	PSR07001802	3.34E-02	-1.32				
	PSR07001803	1.07E-02	-1.44				
	PSR07001848	2.03E-02	1.42				
	PSR07001856	1.80E-02	1.48				
IMPA2	JUC18000387	1.79E-02	-1.87				
ITGA2B	JUC17011930	3.93E-02	-2.90				
	JUC17011947	4.46E-02	-3.17				
	JUC17011952	1.06E-02	-3.94				
	PSR17021296	4.53E-02	-1.45				
	PSR17021297	3.03E-02	-2.94				
	PSR17021298	3.14E-02	-1.81				
	PSR17021300	2.34E-02	-2.84				
	PSR17021301	3.10E-02	-2.68				
	PSR17021306	3.60E-02	-2.49				
ITGB3	JUC17018703	3.77E-02	-3.20				
	JUC17018705	1.67E-02	-2.85				
	JUC17018708	1.28E-02	-4.16				
	JUC17018710	2.87E-02	-2.82				
	JUC17018711	2.07E-02	-3.26				
	JUC17018712	1.97E-02	-3.72				
	JUC17018713	2.24E-02	-4.07				
	JUC17018714	2.81E-03	-5.12				
	JUC17018715	2.13E-02	-3.41				

	JUC17018719					6.30E-04	1.21
	JUC17018725					8.04E-04	1.20
	JUC17018727					3.68E-02	1.52
	JUC17018731					2.16E-04	1.65
	JUC17018740					3.66E-02	-1.43
	JUC17018745					2.14E-02	1.48
	JUC17018749			2.18E-02	1.96		
	JUC17018751			4.88E-02	2.01		
	PSR17007417	2.78E-02	-3.08				
	PSR17007419	2.83E-02	-2.70				
	PSR17007420	2.19E-02	-3.12				
	PSR17007421	2.41E-02	-3.20				
	PSR17007428	3.52E-02	-2.84				
	PSR17007429	2.81E-02	-2.95				
	PSR17007431	3.64E-02	-2.82				
	PSR17007433	2.55E-02	-2.97				
	PSR17007445			1.86E-02	1.85		
	PSR17007452			4.56E-02	1.62		
	PSR17007457			1.27E-02	1.74		
	PSR17007459			5.09E-03	2.59	4.78E-02	1.37
	PSR17007460			4.68E-02	1.47		
	PSR17007461			1.46E-03	1.55		
	PSR17007462			3.13E-02	2.31	1.96E-02	1.61
	PSR17007464					2.66E-02	1.48
	PSR17007469			3.94E-02	1.94		
	PSR17007472					1.07E-02	1.49
	PSR17007473	4.94E-02	1.28				
	PSR17007480			2.79E-02	1.59		
	PSR17007481					2.05E-02	1.32
	PSR17007482	4.56E-02	1.28	4.59E-02	1.42		
LDLR	JUC19001721					3.22E-02	-1.34
	JUC19001728	2.08E-02	-1.32				
	PSR19002958	4.01E-02	-1.63				
	PSR19002980	4.38E-02	-1.22				
LPA	JUC06014309	3.83E-02	1.38				
	JUC06014314					2.20E-02	-1.43
	PSR06028654	6.81E-03	1.37				
	PSR06028656	6.81E-03	1.37				
	PSR06028658	6.81E-03	1.37				
	PSR06028660	6.81E-03	1.37				
	PSR06028662	6.81E-03	1.37				
	PSR06028664	6.81E-03	1.37				
	PSR06028666	6.81E-03	1.37				
LPAL2	PSR06028620			4.71E-02	-2.18		
LPL	JUC08000805	5.00E-02	1.37				
MTHFR	JUC01018277	3.06E-02	-1.98				
	JUC01018283	3.48E-02	-1.52	1.18E-02	2.32		
	JUC01018285			4.42E-02	1.79		
	PSR01034067			3.33E-02	1.35		
	PSR01034075	1.67E-02	-1.54				

NAA25	JUC12014408					2.83E-02	1.57
	JUC12014412			2.35E-02	-1.40		
	JUC12014416	1.48E-02	-1.41				
	JUC12014426			4.48E-02	-1.42		
	JUC12014429					2.97E-02	-1.42
NINJ2	JUC12007875					3.32E-02	1.94
	PSR12014329					2.07E-02	1.90
	PSR12014331					2.69E-02	1.68
	PSR12014332					3.34E-02	1.80
	PSR12014335					2.45E-02	1.65
	PSR12014336					2.50E-02	1.71
NOS1	JUC12014768					7.77E-03	-1.73
	JUC12014792					1.93E-02	-1.42
NOS3	JUC07007452			4.15E-02	-1.92		
	JUC07007467					2.04E-03	-1.82
	JUC07007474					4.63E-02	-1.43
	JUC07007479	4.57E-02	1.26				
	PSR07015406					3.10E-02	-1.38
	PSR07015446	3.54E-02	1.34				
PCSK9	JUC01005610					3.18E-02	1.82
PDE4D	JUC05010008			3.24E-02	1.28		
	PSR05019351			2.62E-02	-2.02		
	PSR05019355	2.40E-02	-1.48				
	PSR05019360	4.00E-02	-1.30				
	PSR05019367			7.08E-03	1.52		
	PSR05019383	3.79E-02	-1.89				
	PSR05019391			3.22E-03	1.62		
	PSR05019409	1.83E-02	1.28				
	PSR05019416	4.71E-02	1.37			1.73E-02	-1.37
	PSR05019421	2.93E-02	1.33				
PDGFC	JUC04012328	1.94E-02	1.47				
	JUC04012332			3.21E-02	1.52		
PEMT	JUC17009234	4.68E-02	1.62				
	PSR17016158					3.04E-03	1.43
	PSR17016167					1.53E-02	1.48
	PSR17016172					3.87E-02	1.42
PITX2	PSR04020916					3.03E-02	-1.28
PLPP3	PSR01042375					4.78E-02	-1.71
PON1	JUC07021478	1.97E-03	1.90				
PRKCH	JUC14002035	5.37E-03	-1.56				
	JUC14002036	1.38E-02	-2.28				
	JUC14002038	1.02E-02	-1.79				
	JUC14002039	1.47E-02	-1.71				
	JUC14002043	1.62E-02	-2.30				
	JUC14002046	2.26E-02	-2.30				
	JUC14002048	1.53E-02	-1.62				
	JUC14002049	4.11E-02	-1.76				
	JUC14002050	2.83E-02	-1.53				
	PSR14004093	5.34E-03	-1.89	4.07E-02	-2.04		
	PSR14004094	3.87E-02	-1.78				

	PSR14004095	3.18E-02	-1.48				
	PSR14004097	4.22E-02	-1.48				
	PSR14004098	1.17E-02	-2.06				
	PSR14004099	5.04E-03	-1.93				
	PSR14004100	2.15E-03	-2.01	4.56E-02	-1.98		
	PSR14004105	4.94E-02	-1.52				
	PSR14004107	4.08E-03	-1.58	4.00E-02	-1.67		
RAI1	JUC17001562					3.54E-02	-1.72
SGK1	JUC06013221	4.21E-02	-2.15				
	PSR06026696	3.43E-02	-1.42				
	PSR06026705	2.39E-02	-1.49				
	PSR06026707	3.13E-02	-1.47				
	PSR06026708	3.13E-02	-1.42				
	PSR06026710	4.51E-02	-1.41				
	PSR06026714					4.60E-02	1.27
	PSR06026719	3.74E-02	-1.84				
SH2B3	JUC12006317					3.92E-02	-1.34
	PSR12011380	1.91E-02	-1.31				
**SLC22A3**	JUC06006832					4.42E-03	-1.40
SLC4A1	JUC17011885					5.44E-03	1.43
	JUC17011886					3.36E-02	1.66
	JUC17011893					1.30E-02	1.72
	PSR17021220					5.21E-04	2.26
SMARCA4	JUC19001671	3.50E-02	1.62				
	JUC19001675					3.58E-02	-1.67
	JUC19001678					2.62E-02	1.55
	JUC19001688					2.46E-06	1.96
	JUC19001690					1.67E-02	-1.51
	JUC19001697					2.78E-02	-1.44
	JUC19001698	1.55E-02	-1.82				
	JUC19001699	1.86E-02	-1.72				
	JUC19001703					3.82E-02	1.68
	JUC19001709	3.32E-02	-1.84				
	JUC19001711					1.03E-02	1.38
	PSR19002909	2.98E-02	-1.46				
	PSR19002936					3.32E-02	1.28
	PSR19002940					4.87E-02	1.37
	PSR19002941					4.84E-02	1.68
	PSR19002942					4.10E-02	1.52
SORT1	JUC01024735	3.82E-02	-2.74				
	JUC01024751	3.13E-02	1.71			7.07E-03	-1.73
	PSR01045986	6.93E-03	-1.35				
WNK1	JUC12000091					5.18E-03	1.92
	JUC12000093					2.17E-02	1.49
	JUC12000095					2.58E-02	1.99
	JUC12000096					4.45E-03	2.63
	JUC12000097					7.09E-03	1.67
	JUC12000098					9.18E-03	1.50
	JUC12000100					1.05E-03	1.93
	JUC12000101					1.31E-02	1.54

	JUC12000102					6.49E-03	1.76
	JUC12000103					4.99E-02	-1.97
	JUC12000105					5.75E-03	2.39
	JUC12000106			4.81E-02	-1.68		
	JUC12000111					8.66E-03	1.87
	JUC12000112					3.01E-03	3.12
	JUC12000118					4.83E-03	1.68
	JUC12000119					5.69E-03	1.71
	JUC12000121					1.02E-02	2.40
	JUC12000125					1.86E-02	2.10
	JUC12000127					3.42E-03	1.74
	JUC12000128					4.21E-03	2.03
	JUC12000129					5.69E-03	1.41
	JUC12000130					1.34E-02	1.43
	JUC12000131					9.44E-04	1.84
	JUC12000132					1.22E-02	2.00
	PSR12000128					1.72E-03	1.91
	PSR12000131					1.38E-03	1.82
	PSR12000135			3.61E-02	-1.73	1.93E-02	1.41
	PSR12000136			4.08E-02	-1.64	1.34E-02	1.38
	PSR12000137					1.20E-02	1.43
	PSR12000140	3.29E-02	-1.26				
	PSR12000141					1.76E-02	1.46
	PSR12000143					3.34E-02	1.40
	PSR12000144					1.21E-02	1.47
	PSR12000145					1.15E-02	1.46
	PSR12000147					9.32E-03	1.57
	PSR12000148					5.86E-03	1.62
	PSR12000151					1.60E-02	1.57
	PSR12000153					5.08E-03	1.71
	PSR12000155					4.22E-03	1.99
	PSR12000156					8.77E-03	1.92
	PSR12000157					6.28E-03	1.75
	PSR12000158					9.04E-03	1.67
	PSR12000159					3.08E-03	1.69
	PSR12000160					2.17E-03	1.74
	PSR12000161					1.91E-03	1.70
	PSR12000162					2.59E-03	1.88
	PSR12000163					7.72E-03	2.01
	PSR12000164					6.20E-03	2.43
	PSR12000165					6.95E-03	1.69
	PSR12000167					1.65E-03	2.07
	PSR12000168					4.37E-03	1.99
	PSR12000169					1.46E-02	1.58
	PSR12000170					1.36E-02	1.78
	PSR12000172			1.04E-02	-1.93		
	PSR12000173					1.77E-03	1.61
	PSR12000174					1.27E-02	1.76
	PSR12000175					9.20E-03	1.63
	PSR12000176					1.19E-02	1.57
	PSR12028411					1.42E-02	1.86

WNK1-lncRNA	PSR12028412			3.35E-02	-1.69	4.22E-02	-1.28
	PSR12028413					8.09E-03	1.52
ZC3HC1	PSR07028794					4.56E-02	1.49
ZFHX3	JUC16009969			2.60E-02	-1.84		
ZPR1	JUC11014794	2.28E-02	1.28				
	JUC11014802					2.89E-02	1.44

Supplementary Table 4. Genes represented by exons/junctions significant for time in hours since event in male and female ischemic stroke patients. Significance: $p<0.05$; correlation, $r>|0.4|$. Three junctions (PDE4D, NOS3, LDLR) and 1 within exon probeset common between sexes are bolded in the table below. Italicized common probesets (LDLR and APOE) differ in partial correlation direction.

Gene Symbol	Exon (PSR) and Junction (JUC) Probesets	Male (n=40)		Female (n=45)	
		p-value	r	p-value	r
ABO	PSR09021554			4.09E-02	-0.41
ACE	JUC17005476	1.71E-02	-0.45		
	PSR17009933	2.26E-02	-0.43		
	PSR17009950	2.91E-02	-0.41		
	PSR17009953	9.72E-03	-0.48		
	PSR17009958			4.63E-03	-0.55
ADD1	PSR04000990			1.71E-02	-0.47
ALDH2	JUC12019437	2.50E-02	-0.42		
	JUC12019453	1.54E-02	0.45		
	JUC12019456			3.65E-02	0.42
	PSR12011414	2.47E-02	0.42		
	PSR12011418	3.21E-02	0.41		
	PSR12011429	4.00E-03	0.53		
	PSR12011493	2.13E-02	-0.43		
ANGPT1	JUC08010140			2.80E-02	-0.44
	JUC08010143			1.12E-02	-0.50
	JUC08010144			7.60E-03	-0.52
	JUC08010147			3.42E-04	-0.66
	PSR08019927			2.16E-02	-0.46
	PSR08019929			1.60E-05	-0.75
	PSR08019931			2.51E-02	-0.45
	PSR08019935			9.44E-03	-0.51
	PSR08019940			3.22E-02	-0.43
	PSR08019942			3.62E-02	-0.42
	PSR08019947			4.23E-02	-0.41
	PSR08019948			1.88E-02	-0.47
	PSR08019952	1.76E-02	-0.45		
APOE	PSR19008804			4.41E-02	-0.41
	PSR19008805			3.54E-02	-0.42
	PSR19008811	***3.42E-02***	***-0.40***	***2.63E-02***	***0.44***

CDC5L	JUC06003325	2.35E-02	-0.43		
CDKN2A	PSR09012762			3.46E-02	-0.42
	PSR09012770			4.42E-02	-0.41
CDKN2B	JUC09006957			9.18E-04	-0.62
	PSR09012792	1.34E-02	-0.46		
CRP	PSR1052888			2.84E-02	-0.44
	PSR01052891	1.61E-02	-0.45		
CYP11B2	JUC08011555			4.34E-02	0.41
CYP4A11	JUC01021982	3.18E-03	0.54		
	JUC01021984			2.29E-02	-0.45
	JUC01021985			2.96E-02	-0.44
DDAH1	JUC01023612			2.06E-02	-0.46
	JUC01023617			1.22E-02	0.49
	JUC01023622			4.19E-02	0.41
	JUC01023626			1.80E-02	-0.47
	PSR01044037			4.20E-02	-0.41
EPHX2	JUC08001477			2.92E-02	-0.44
	JUC08001483			7.20E-04	0.63
	JUC08001488			4.52E-02	0.40
	PSR08003024			1.91E-02	0.47
F13A1	JUC06007366			2.64E-02	0.44
F13B	JUC01029317			4.32E-02	-0.41
F2	PSR11005072	2.17E-02	-0.43		
	PSR11005084	8.75E-03	-0.49		
F5	JUC01028347			2.06E-02	0.46
F7	PSR13004634	3.10E-02	-0.41		
FGA	JUC04012257	3.06E-02	-0.41		
	PSR04023166	3.02E-02	-0.41		
FGB	JUC04005757			1.26E-02	-0.49
	JUC04005758	6.40E-03	-0.50		
	JUC04005764			6.91E-03	-0.53
	JUC04005766			6.49E-03	-0.53
	PSR04010731			4.35E-02	-0.41
GP1BA	PSR17000619	2.02E-02	0.44		
HABP2	JUC10005522			2.98E-02	-0.43
	JUC10005530	2.52E-02	-0.42		
	PSR10009904	1.40E-02	-0.46		
	PSR10009907	6.67E-03	-0.50		
HDAC9	JUC07000793			3.63E-02	-0.42
	JUC07000795			7.19E-03	0.52
	JUC07000797			9.66E-04	-0.62
	JUC07000818			2.22E-02	0.46
	JUC07000820	2.80E-03	-0.54		
	PSR07001772			1.16E-02	0.50
	PSR07001777	3.17E-02	-0.41		
IL1A	PSR02036041	3.27E-02	-0.40		
IL6	JUC07001034			2.26E-02	-0.45

ITGB3	JUC17018702			4.10E-03	-0.55
	JUC17018735			2.91E-02	0.44
	PSR17007424			3.67E-02	-0.42
	PSR17007437			2.94E-02	-0.44
	JUC17018756	6.36E-03	-0.50		
	PSR17007451	3.77E-03	0.53		
**LDLR**	***JUC19001725***	***2.90E-02***	***-0.41***	***4.39E-02***	***0.41***
	JUC19001734			1.64E-02	0.47
	PSR19002973			3.30E-02	0.43
LPA	JUC06014349	4.55E-03	-0.52		
	JUC06014355	4.55E-03	-0.52		
	JUC06014360	4.55E-03	-0.52		
	JUC06014364	4.55E-03	-0.52		
	JUC06014366	4.55E-03	-0.52		
	JUC06014367	3.29E-03	-0.54		
	JUC06014374	3.29E-03	-0.54		
	PSR06028635	2.31E-02	-0.43		
	PSR06028641			3.17E-02	0.43
LPL	JUC08000808	2.90E-02	-0.41		
	PSR08001517	1.23E-02	-0.47		
	PSR08001520	6.95E-03	-0.50		
	PSR08001528	1.28E-02	-0.46		
LTA	PSR06003535			9.45E-03	0.51
LTC4S	PSR05015386			1.90E-02	-0.47
MMP12	PSR11026742	2.76E-02	-0.42		
MTHFR	JUC01018274			2.34E-03	0.58
	JUC01018276	3.00E-02	0.41		
NINJ2	PSR12014337	2.38E-02	0.43		
NOS1	JUC12014760			2.39E-02	-0.45
	JUC12014764	5.12E-03	-0.51		
	JUC12014767	2.38E-02	-0.43		
	JUC12014769	2.91E-02	0.41		
	JUC12014777	1.93E-02	-0.44		
	PSR12026417			2.16E-02	0.46
	PSR12026420	2.38E-02	-0.43		
**NOS3**	JUC07007447	3.49E-02	0.40		
	JUC07007452	1.22E-02	-0.47		
	JUC07007458			8.68E-04	-0.62
	JUC07007459			2.17E-02	-0.46
	JUC07007461			3.14E-02	-0.43
	**JUC07007469**	**2.16E-02**	**-0.43**	**3.37E-02**	**-0.43**
	JUC07007472	2.24E-03	0.55		
	PSR07015412			3.16E-02	-0.43
	PSR07015428			3.04E-03	-0.57
	PSR07015439			1.83E-02	0.47
PCSK9	JUC01005617			2.36E-02	-0.45
	PSR01010956			3.42E-03	0.56

	PSR01010958	2.34E-02	0.43		
**PDE4D**	JUC05009957	1.01E-02	-0.48		
	JUC05009961			9.10E-03	0.51
	**JUC05009966**	**2.46E-02**	**-0.42**	**2.25E-02**	**-0.45**
	JUC05009968	2.59E-03	0.55		
	JUC05010000	3.11E-03	0.54		
	JUC05010010	2.68E-02	0.42		
	JUC05010011	1.52E-02	0.45		
	PSR05019335	1.89E-02	0.44		
	PSR05019339	2.95E-02	0.41		
	PSR05019373	2.02E-03	0.56		
	PSR05019374	4.91E-03	0.52		
	PSR05019376	1.48E-02	0.46		
	PSR05019377	1.83E-02	0.44		
	PSR05019403	2.65E-02	0.42		
	PSR05019418	1.64E-02	0.45		
	PSR05019422	3.62E-03	-0.53		
	PSR05019423			3.92E-02	0.41
PDGFC	JUC04012329			1.79E-02	-0.47
	PSR04023294			4.20E-02	-0.41
	PSR04023296			2.60E-02	-0.44
	PSR04023305			9.00E-03	-0.51
	PSR04023314	3.40E-02	-0.40		
	PSR04023316			1.83E-02	0.47
	PSR04023317			1.52E-02	-0.48
	PSR04023322	2.98E-02	-0.41		
	PSR04029574			4.84E-06	-0.78
PITX2	JUC04010976	2.49E-02	-0.42		
	JUC04010977	3.32E-02	-0.40		
	PSR04020906			1.96E-02	0.46
	PSR04020915	2.21E-02	-0.43		
	PSR04020917	3.04E-02	-0.41		
PLPP3	JUC01022662			4.20E-02	-0.41
PON1	JUC07021472			2.64E-02	-0.44
	PSR07024534			3.57E-02	-0.42
	PSR07024525	1.16E-02	-0.47		
	PSR07024532	3.12E-02	0.41		
PTGIS	JUC20006655			4.31E-02	-0.41
	JUC20006658			3.53E-02	-0.42
	PSR20012768	3.45E-02	-0.40		
RAI1	JUC17001545	1.24E-02	-0.47		
	JUC17001561	1.89E-02	-0.44		
	PSR17002770	3.50E-03	0.53		
	PSR17002786			4.62E-02	-0.40
	PSR17002791			2.33E-02	-0.45
SERPINE1	JUC07004647			3.44E-02	-0.42
SGK1	JUC06013222			2.65E-02	-0.44

	JUC06013238	2.84E-02	-0.41		
	PSR06026704	2.72E-02	0.42		
	PSR06026742			7.08E-04	-0.63
SH2B3	PSR12011375			4.06E-02	-0.41
SLC22A3	JUC06006831			3.24E-02	-0.43
	JUC06006832			3.16E-02	0.43
	JUC06006837			2.72E-02	-0.44
	PSR06013800	3.14E-02	-0.41		
SMARCA4	JUC19001688			3.40E-02	-0.43
	JUC19001714			1.31E-02	-0.49
	PSR19002898	9.38E-03	-0.48		
SUPT3H	JUC06010105	2.90E-02	0.41		
	PSR06021220	1.05E-02	0.48		
	PSR06021221	1.24E-02	0.47		
	PSR06021232	1.78E-02	0.44		
	PSR06021243			4.43E-02	0.41
ZC3HC1	JUC07014244			4.29E-02	-0.41
	JUC07014254	2.86E-02	-0.41		
	PSR07028810			3.30E-03	-0.56
ZFHX3	JUC16009976	1.95E-02	0.44		
	PSR16017564	3.87E-03	-0.53		
ZPR1	JUC11014803			4.24E-02	0.41
	JUC11014805	1.03E-02	-0.48		
